# Supplementary material for: Evidence for the early emergence of piperaquine-resistant Plasmodium falciparum malaria and modeling strategies to mitigate resistance
Source: PLoS Pathog. 2022 Feb 7;18(2):e1010278. doi: 10.1371/journal.ppat.1010278 (PMC8853508; doi:10.1371/journal.ppat.1010278)
Supplement: S1 Fig — (A) The pfcrt gene was edited using a two-plasmid approach, one containing the donor and the other expressing the pfcrt-specific ZFN. Parasites were selected on WR99210 and blasticidin (BSD) and cloned by limiting dilution. (B) Three sets of PCRs were performed to confirm editing and the modified locus was verified by Sanger sequencing. Primer sequences are noted in S9 Table. (PDF) [file ppat.1010278.s001.pdf]

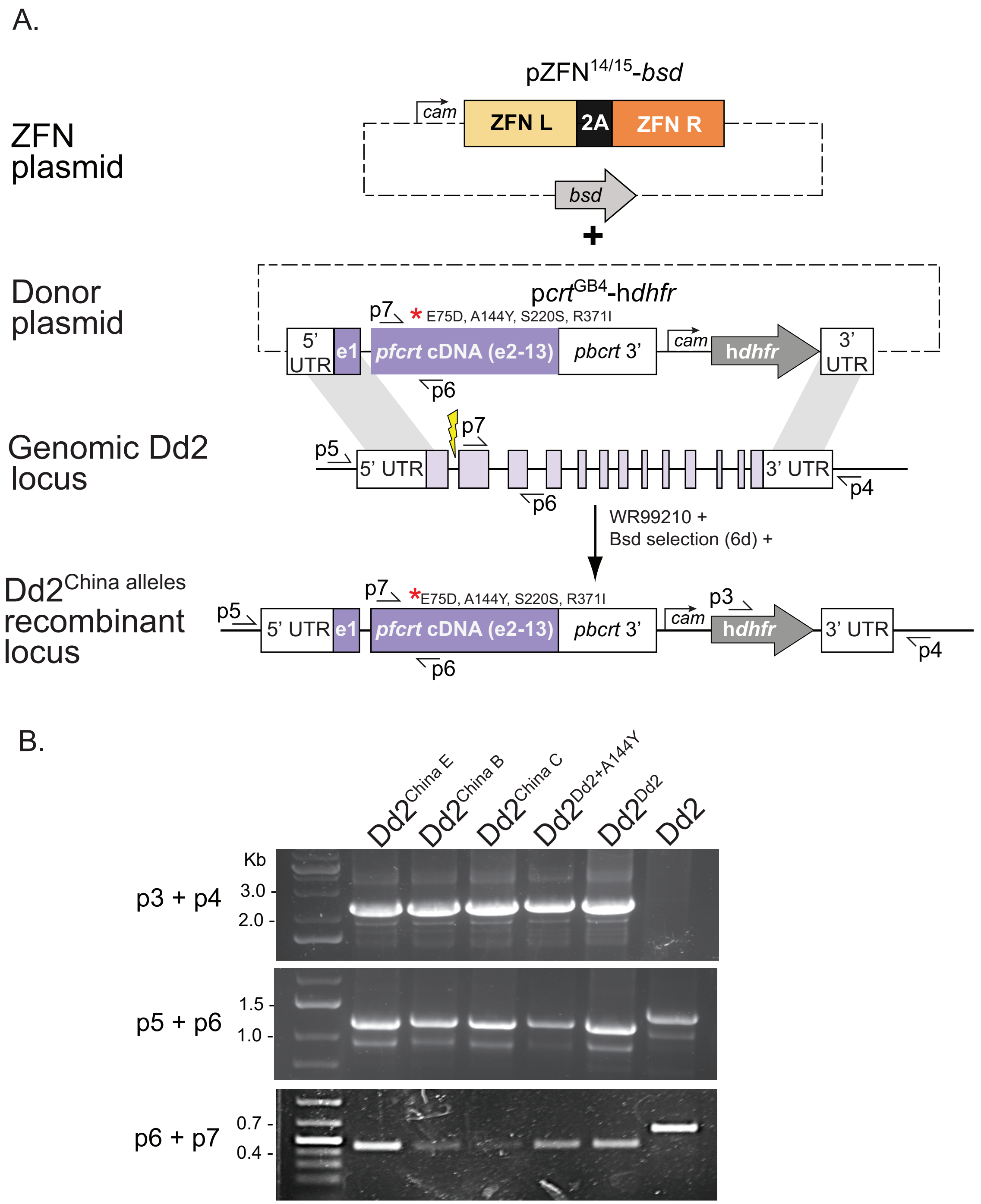

S1 Fig. Zinc-Finger Nuclease (ZFN)-mediated editing of *pfcrt.* (A) The *pfcrt* gene was edited using a two-plasmid approach, one containing the donor and one containing the *pfcrt*-specific ZFN. Parasites were selected on WR99210 and blasticidin (BSD) and cloned by limiting dilution. **(B)** Three sets of PCRs were performed to confirm editing and the modified locus was verified by Sanger sequencing. Primer sequences are noted in **S9 Table**.
