# Supplementary material for: Evidence for the early emergence of piperaquine-resistant Plasmodium falciparum malaria and modeling strategies to mitigate resistance
Source: PLoS Pathog. 2022 Feb 7;18(2):e1010278. doi: 10.1371/journal.ppat.1010278 (PMC8853508; doi:10.1371/journal.ppat.1010278)
Supplement: S2 Fig — Mean ± SEM (A) IC50 values and (B) IC90 values were determined by conventional 72-hr dose-response assays performed with asynchronous parasite cultures. N, n = 5, 2. PPQ IC90 values could not be calculated for the Dd2Dd2+F145I line because of the biphasic nature of its dose-response curve. Statistical significance was determined via two-tailed Mann-Whitney U tests as compared to the isogenic line. *P<0.05, ** P <0.01. Values are noted in S2 Table. (PDF) [file ppat.1010278.s002.pdf]

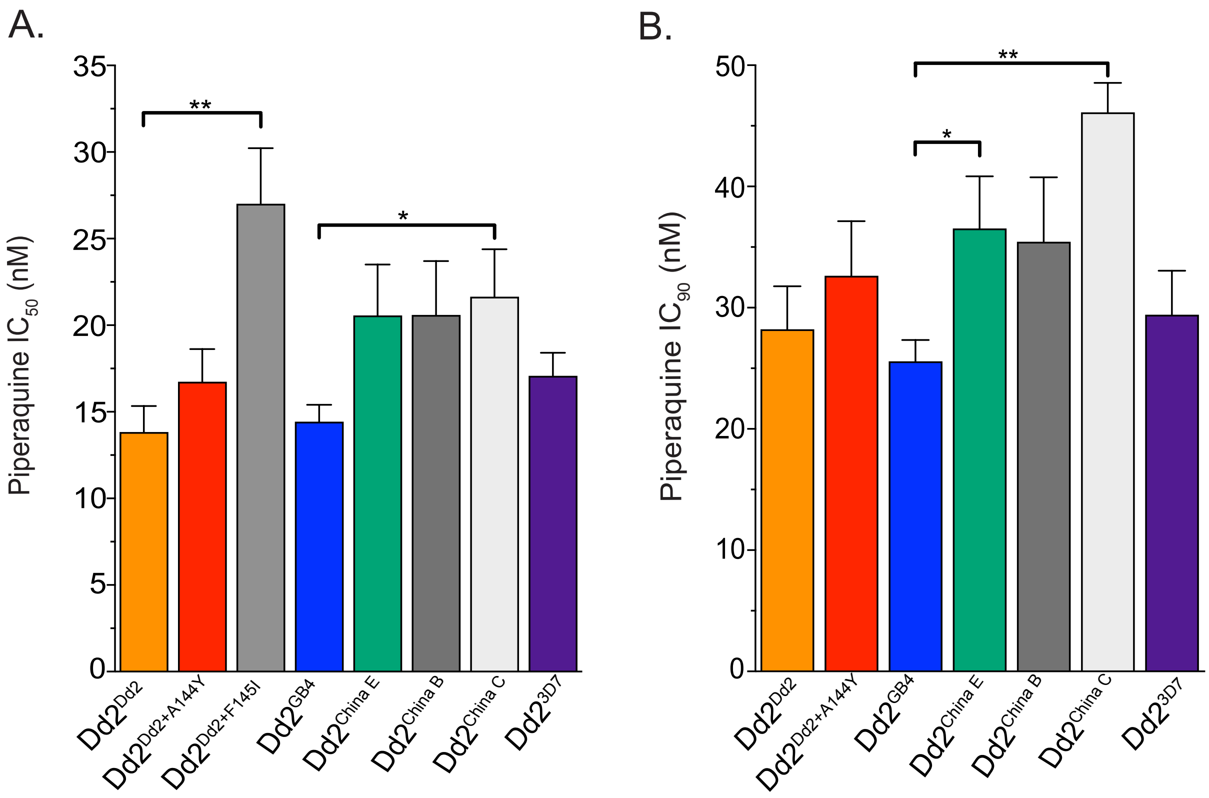

S2 Fig. Piperaquine dose-response data. Mean ± SEM (**A**) IC_{50} values and (**B**) IC_{90} values were determined by conventional 72-hr dose-response assays performed with asynchronous parasite cultures. *N*, *n* = 5, 2. PPQ IC_{90} values could not be calculated for the $Dd2^{Dd2+F145I}$ line because of the biphasic nature of its dose-response curve. Statistical significance was determined via two-tailed Mann-Whitney *U* tests as compared to the isogenic line. **P*<0.05, ** *P* <0.01. Values are noted in **S2 Table**.
