# Supplementary material for: Evidence for the early emergence of piperaquine-resistant Plasmodium falciparum malaria and modeling strategies to mitigate resistance
Source: PLoS Pathog. 2022 Feb 7;18(2):e1010278. doi: 10.1371/journal.ppat.1010278 (PMC8853508; doi:10.1371/journal.ppat.1010278)
Supplement: S3 Fig — Dd2China C and to a lesser extent Dd2China B have distended, translucent digestive vacuoles in both the trophozoite and schizont stages. This is not observed in Dd2GB4 or Dd2China E. Dd2Dd2+F145I has the most distended vacuoles in both stages. Of note, the Dd2Dd2 parasites have mildly distended vacuoles in the trophozoite stage only, as previously reported [43]. (PDF) [file ppat.1010278.s003.pdf]

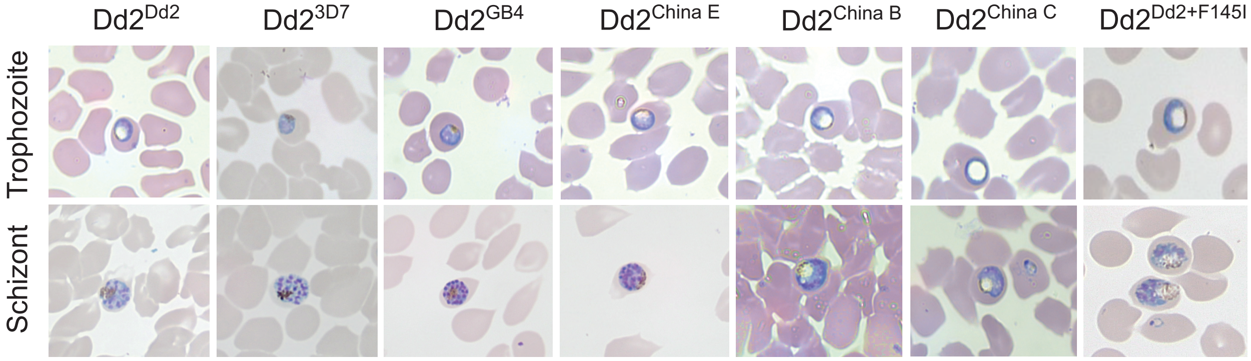

S3 Fig. Cell morphology of *pfcrt*-edited parasites. Dd2$^{China\ C}$ and to a lesser extent Dd2$^{China\ B}$ have distended, translucent digestive vacuoles in both the trophozoite and schizont stages. This is not observed in Dd2^{GB4} or Dd2$^{China\ E}$. Dd2$^{Dd2+F145I}$ has the most distended vacuoles in both stages. Of note, the Dd2^{Dd2} parasites have distended vacuoles in the trophozoite stage only, as reported by Dhingra *et al.* 2017 mBio; PMID: 28487425.
