# Supplementary material for: Evidence for the early emergence of piperaquine-resistant Plasmodium falciparum malaria and modeling strategies to mitigate resistance
Source: PLoS Pathog. 2022 Feb 7;18(2):e1010278. doi: 10.1371/journal.ppat.1010278 (PMC8853508; doi:10.1371/journal.ppat.1010278)
Supplement: S4 Fig — Mean ± SEM IC50 values (S2 Table) were calculated from 72-hr dose-response assays for: (A) quinine; (B) mefloquine; (C) monodesethyl (md)-amodiaquine; (D) dihydroartemisinin; (E) pyronaridine; and (F) lumefantrine. N, n = 4–7, 2. Statistical significance was determined via two-tailed Mann-Whitney U tests as compared to the isogenic line. * P <0.05, ** P <0.01. (PDF) [file ppat.1010278.s004.pdf]

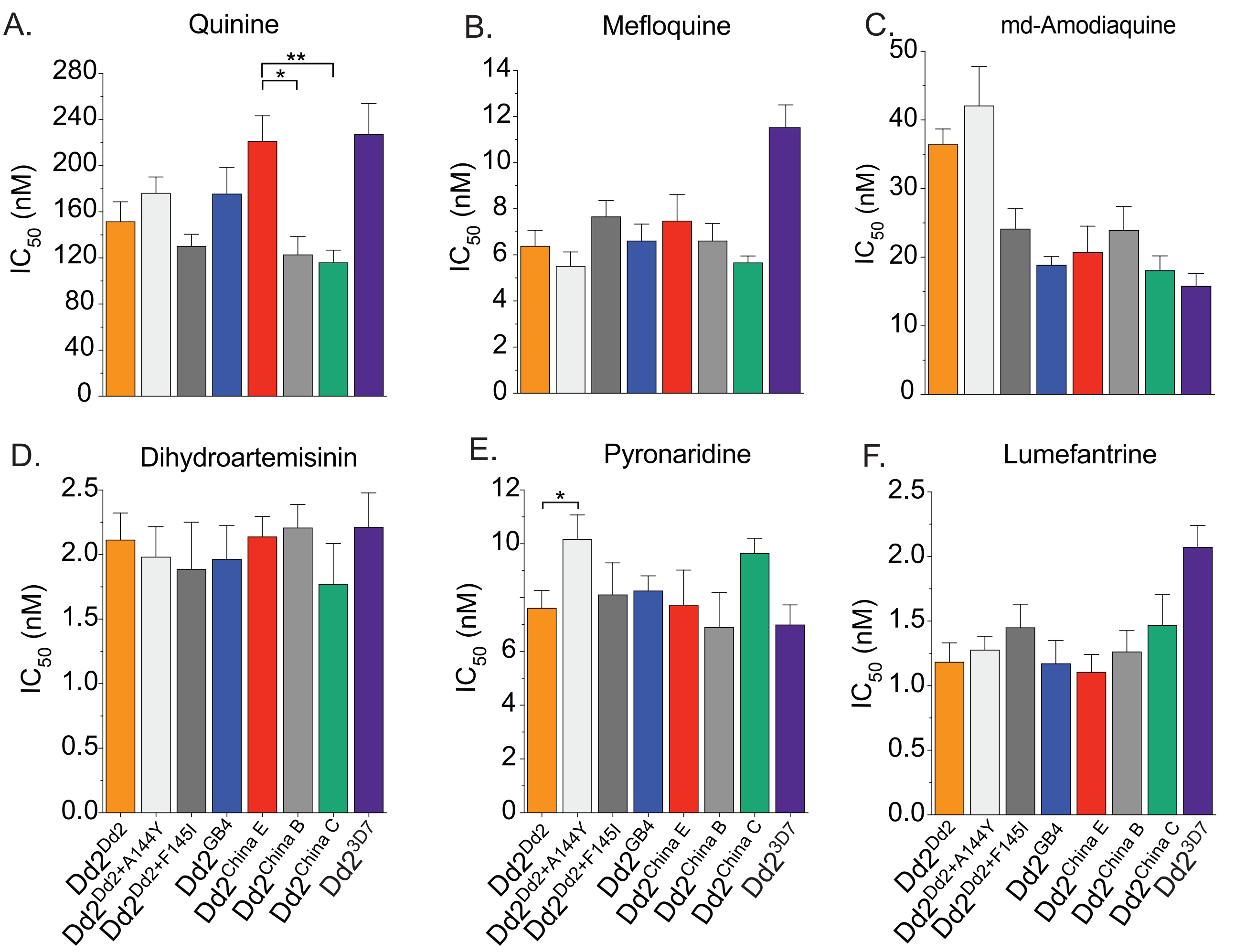

S4 Fig. IC_{50} data for common antimalarials. Mean ± SEM IC_{50} values (**S2 Table**) were calculated from 72-hr dose-response assays for: (**A**) quinine; (**B**) mefloquine; (**C**) monodesethyl (md)-amodiaquine; (**D**) dihydroartemisinin; (**E**) pyronaridine; and (**F**) lumefantrine. *N*, *n* = 4-7, 2. Statistical significance was determined via two-tailed Mann-Whitney *U* tests as compared to the isogenic line. * *P* <0.05, ** *P* <0.01.
