# Supplementary material for: Evidence for the early emergence of piperaquine-resistant Plasmodium falciparum malaria and modeling strategies to mitigate resistance
Source: PLoS Pathog. 2022 Feb 7;18(2):e1010278. doi: 10.1371/journal.ppat.1010278 (PMC8853508; doi:10.1371/journal.ppat.1010278)
Supplement: S5 Fig — (A) Simulations of molecular dynamics on the 7G8 structure (without the PfCRT-specific Fab antibody fragment) with mutations in the China C and China B isoforms modeled over 300-nanosecond (ns) trajectories, establishing the equilibrium positions of protein side chains and distances between position 144 and position 371. (B) Electrostatic surfaces for the solved open-to-digestive-vacuole conformation for the modeled isoforms, predicted at pH 5.0. Images are presented as a vertical slice through the transporter, showing net charges in the cavity and locations of transmembrane (TM) helices. Areas in red represent higher electron density (greater negative charge) while those in blue signify lower electron density. The row below shows the view rotated by 180° relative to the top row. (PDF) [file ppat.1010278.s005.pdf]

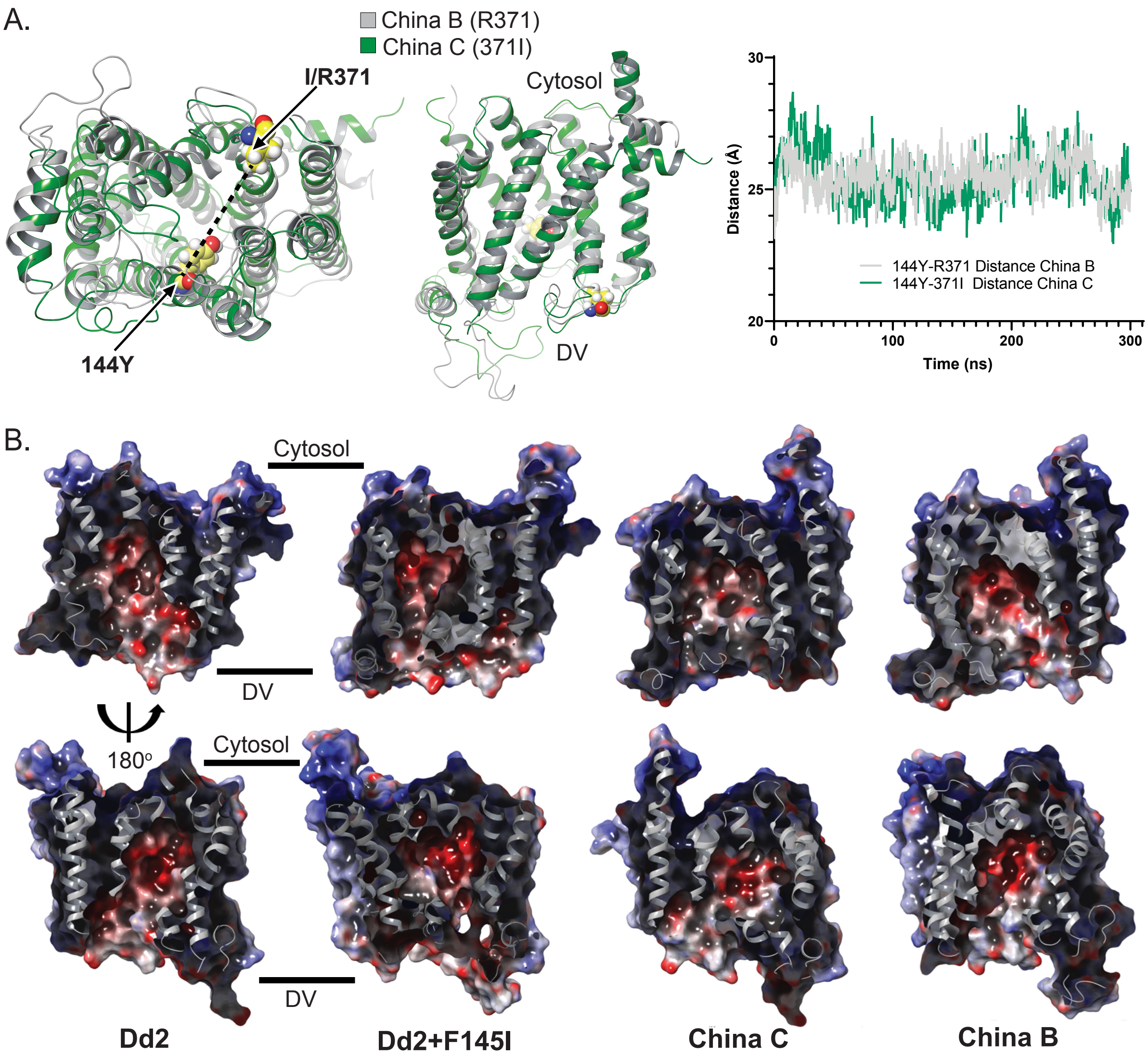

S5 Fig. Simulations of molecular dynamics and electrostatic potential surfaces of isoform-specific PfCRT cavities. (**A**) Simulations of molecular dynamics on the 7G8 structure (without the Fab) with mutations in the China C and China B isoforms modeled over 300-nanosecond (ns) trajectories, establishing the equilibrium positions of protein side chains and distances between position 144 and position 371. (**B**) Electrostatic surfaces for the solved open-to-digestive-vacuole conformation for the modelled isoforms, predicted at pH 5.0. Images are presented as a vertical slice through the transporter, showing net charges in the cavity and locations of transmembrane (TM) helices. Areas in red represent higher electron density (greater negative charge) while those in blue signify lower electron density. The row below shows the view rotated by 180° relative to the top row.
