# Supplementary material for: Evidence for the early emergence of piperaquine-resistant Plasmodium falciparum malaria and modeling strategies to mitigate resistance
Source: PLoS Pathog. 2022 Feb 7;18(2):e1010278. doi: 10.1371/journal.ppat.1010278 (PMC8853508; doi:10.1371/journal.ppat.1010278)
Supplement: S6 Fig — Values on the y-axes are normalized relative to the 24-hr growth rates in the absence of drug (r0). Measurements represent the average of measured data points (see S1 Text). Curves were fitted using the 5-parameter asymmetric Richards equation provided in GraphPad Prism 8 software, with lower and upper bounds constrained to 0 and 1, with the exception that Dd2Dd2+F145I growth under high PPQ concentrations was fit with a quadratic. The simulator uses the fitted curves (with coefficients shown in S4 Table) to interpolate relative growth rates at any desired concentration (in nM) and then multiplies them by the appropriate r0 to infer absolute 24-hr growth rates for a given strain at a given concentration. (PDF) [file ppat.1010278.s006.pdf]

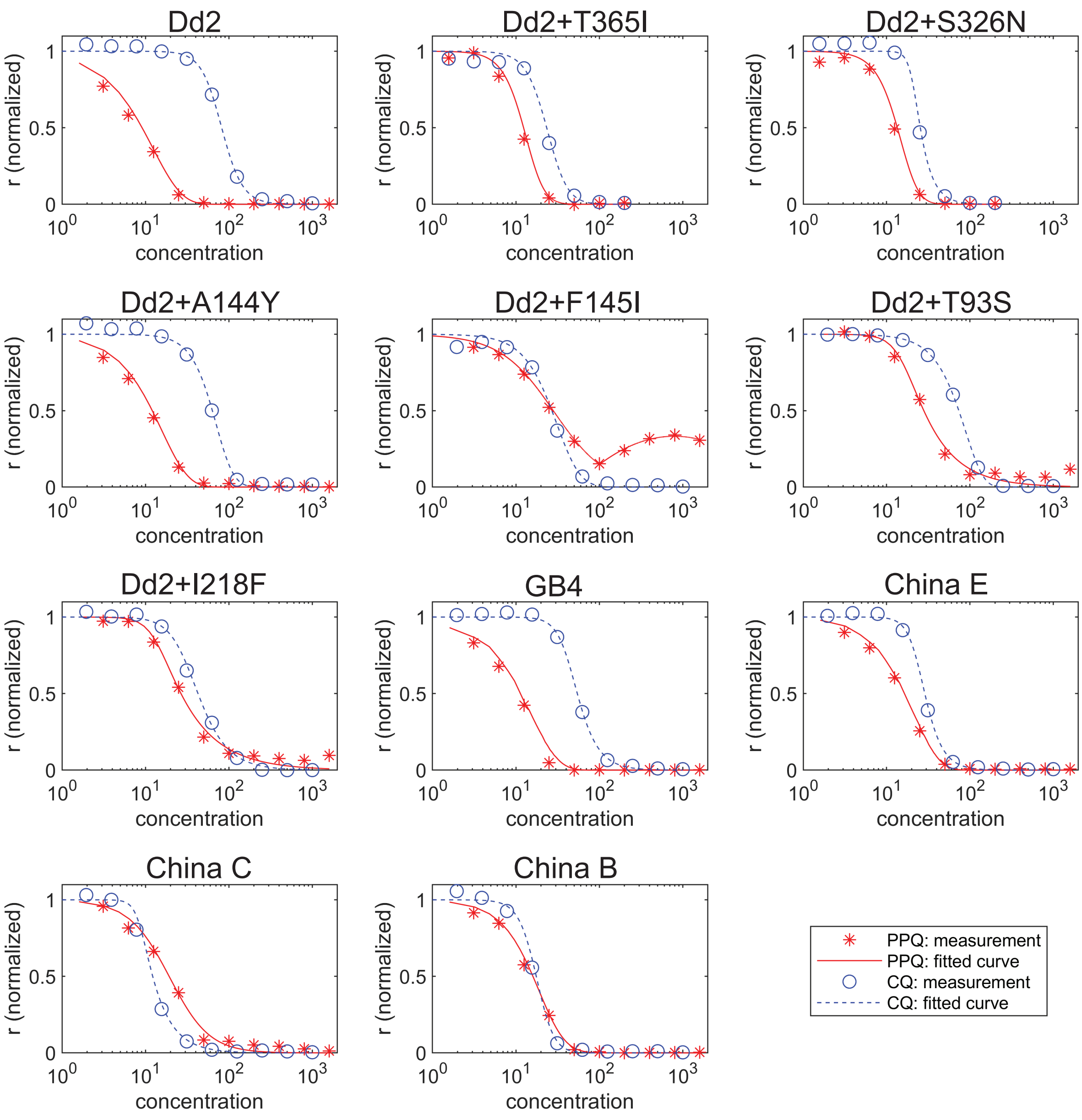


**S6 Fig. Dose response curves for piperaquine (PPQ) and chloroquine (CQ) for the strains included in the study.** Values on the y-axes are normalized relative to the 24-hr growth rates in the absence of drug ($r_0$). Measurements represent the average of measured data points (see Materials and Methods), curves were fitted using the 5-parameter asymmetric Richards equation provided in GraphPad Prism 8 software, with lower and upper bounds constrained to 0 and 1, with the exception that $Dd2^{Dd2+F145I}$ growth under high PPQ concentrations was fit with a quadratic. The simulator uses the fitted curves (with coefficients shown in **S4 Table**) to interpolate relative growth rates at any desired concentration (in nM) and then multiplies them by the appropriate $r_0$ to infer absolute 24-hr growth rates for a given strain at a given concentration.
