# Supplementary material for: Evidence for the early emergence of piperaquine-resistant Plasmodium falciparum malaria and modeling strategies to mitigate resistance
Source: PLoS Pathog. 2022 Feb 7;18(2):e1010278. doi: 10.1371/journal.ppat.1010278 (PMC8853508; doi:10.1371/journal.ppat.1010278)
Supplement: S7 Fig — Representative simulation results starting with infection by a monoculture of PPQ-resistant Dd2Dd2+F145I parasites followed by initial treatment with a low dose regimen of 200 nM PPQ (A) monotherapy; (B, C), late CQ (125 nM) rescue, triggered when the parasite burden again reaches the treatment threshold; or (D, E), simultaneous PPQ and CQ combination treatment. Panels B, D show the most common outcome in 100 stochastic simulations at each treatment regimen. Panels C, E show the next most common outcome. 100 stochastic simulations were run for each condition, with percentage frequencies of a particular outcome shown. Dashed vertical lines indicate when each 3-day dosing began. Strain in this study refers a genetically edited Dd2 parasite with the indicated pfcrt allele. (PDF) [file ppat.1010278.s007.pdf]

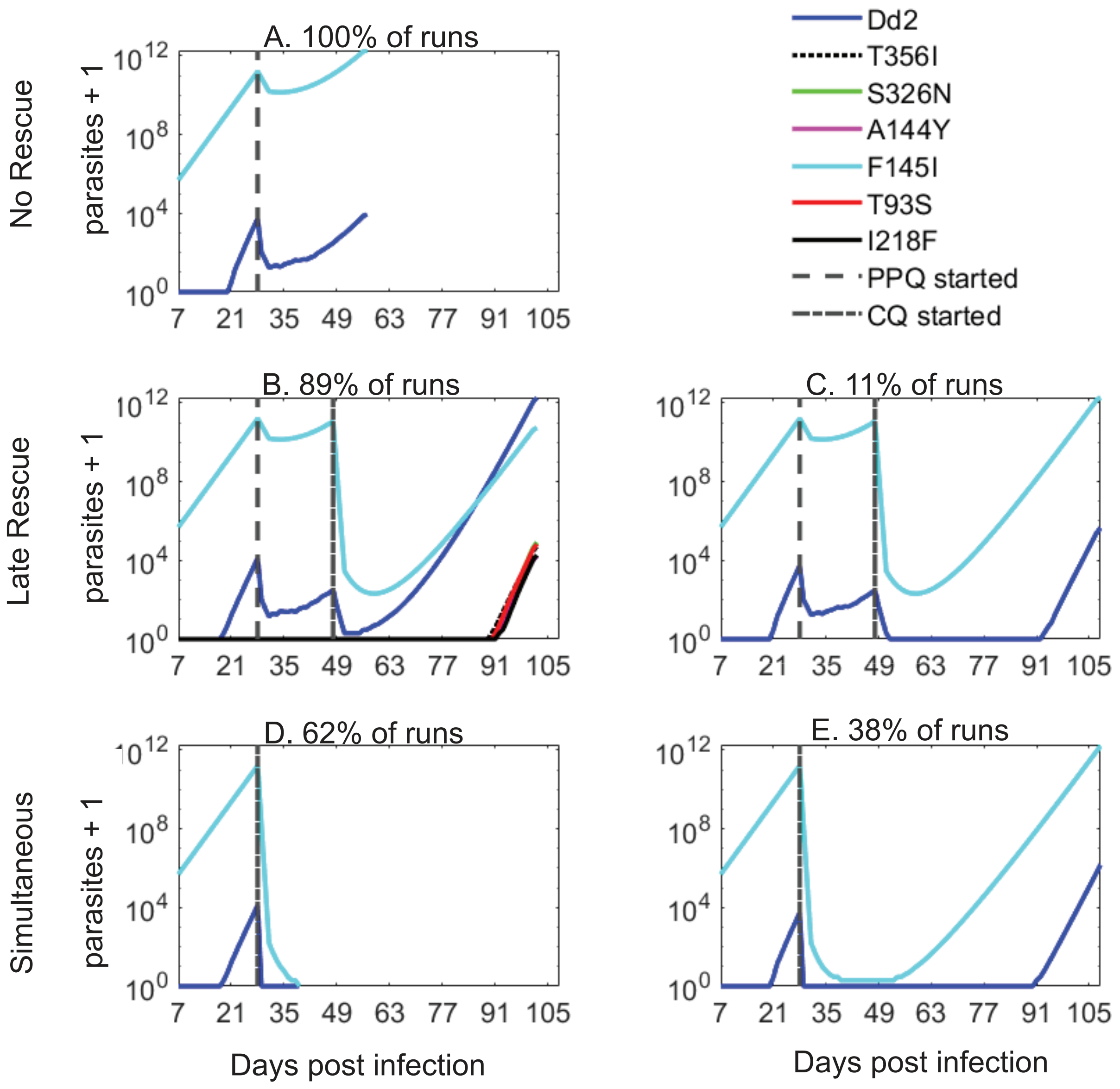

S7 Fig. Evolutionary simulations based upon empirically determined fitness landscapes with a low-dose regimen of piperaquine (PPQ) with or without chloroquine (CQ) in a host infected with the PPQ-resistant Dd2$^{Dd2+F145I}$ strain. Representative simulation results starting with infection by a monoculture of PPQ-resistant Dd2$^{Dd2+F145I}$ parasites followed by initial treatment with a low dose regimen of 200 nM PPQ **(A)** monotherapy; **(B-C)**, late CQ (125 nM) rescue, triggered when the parasite burden again reaches the treatment threshold; or (**D-E)**, simultaneous PPQ and CQ combination treatment. Panels **B, D** show the most common outcome in 100 stochastic simulations at each treatment regime. Panels **C, E** show the next most common outcome. 100 stochastic simulations were run for each condition, with demonstrated percentage frequencies of a particular outcome. Dashed vertical lines indicate when each 3-day dosing began. Strain in this study refers a genetically edited Dd2 parasite with the indicated *pfcrt* allele.
