# Supplementary material for: Evidence for the early emergence of piperaquine-resistant Plasmodium falciparum malaria and modeling strategies to mitigate resistance
Source: PLoS Pathog. 2022 Feb 7;18(2):e1010278. doi: 10.1371/journal.ppat.1010278 (PMC8853508; doi:10.1371/journal.ppat.1010278)
Supplement: S1 Table — (PDF) [file ppat.1010278.s008.pdf]

S1 Table. Piperaquine survival assay values of *pfcrt*-modified parasite lines.

	$Dd2^{Dd2}$	$Dd2^{3D7}$	$Dd2^{GB4}$	$Dd2^{China\ E}$	$Dd2^{China\ B}$	$Dd2^{China\ C}$	$Dd2^{Dd2+F145I}$	$Dd2^{Dd2+A144Y}$
**1600 nM**	**0.79 ± 0.2**	**0.28 ± 0.1**	**0.61 ± 0.2**	**1.4 ± 0.3**	**0.89 ± 0.4**	**2.0 ± 0.5**	**31.4 ± 1.6**	**0.74 ± 0.2**
N	5	5	5	5	5	5	5	5
P vs $Dd2^{Dd2}$	–	0.095	0.548	0.222	>0.999	0.056	0.008	0.841
P vs $Dd2^{GB4}$	0.548	–	–	0.056	0.841	0.032	–	–
P vs $Dd2^{China\ C}$	–	–	–	0.310	0.056	–	–	–
**800 nM**	**0.86 ± 0.2**	**0.26 ± 0.05**	**0.68 ± 0.07**	**1.2 ± 0.3**	**0.76 ± 0.2**	**3.4 ± 0.8**	**34.7 ± 1.0**	**0.96 ± 0.2**
N	5	5	5	5	5	5	5	5
P vs $Dd2^{Dd2}$	–	0.056	0.548	0.421	0.841	0.008	0.008	>0.999
P vs $Dd2^{GB4}$	0.548	–	–	0.151	0.310	0.008	–	–
P vs $Dd2^{China\ C}$	–	–	–	0.032	0.008	–	–	–
**400 nM**	**1.0 ± 0.3**	**0.61 ± 0.3**	**0.45 ± 0.2**	**1.5 ± 0.5**	**0.64 ± 0.3**	**5.0 ± 1.2**	**32.4 ± 3.1**	**1.2 ± 0.4**
N	5	5	5	5	5	5	5	5
P vs $Dd2^{Dd2}$	–	0.310	0.310	0.691	0.421	0.008	0.008	0.841
P vs $Dd2^{GB4}$	0.310	–	–	0.056	0.746	0.008	–	–
P vs $Dd2^{China\ C}$	–	–	–	0.016	0.008	–	–	–
**200 nM**	**1.1 ± 0.3**	**0.22 ± 0.1**	**0.3 ± 0.1**	**1.2 ± 0.2**	**0.8 ± 0.2**	**5.9 ± 1.3**	**24.4 ± 5.5**	**1.6 ± 0.2**
N	5	5	5	5	5	5	5	5
P vs $Dd2^{Dd2}$	–	0.032	0.032	0.691	0.421	0.008	0.008	0.310
P vs $Dd2^{GB4}$	0.032	–	–	0.032	0.032	0.008	–	–
P vs $Dd2^{China\ C}$	–	–	–	0.008	0.008	–	–	–
**100 nM**	**1.3 ± 0.3**	**0.28 ± 0.1**	**0.62 ±0.2**	**1.8 ±0.5**	**1.6 ± 0.5**	**9.8 ± 2.2**	**18.7 ± 4.5**	**3.0± 0.6**
N	6	6	6	6	6	6	6	6
P vs $Dd2^{Dd2}$	–	0.004	0.219	0.457	0.567	0.002	0.002	0.087
P vs $Dd2^{GB4}$	0.219	–	–	0.058	0.217	0.002	–	–
P vs $Dd2^{China\ C}$	–	–	–	0.002	0.002	–	–	–
**50 nM**	**1.8 ± 0.3**	**1.1 ± 0.6**	**0.52 ± .2**	**4.4 ± 2.4**	**3.0 ± 0.7**	**9.3 ± 2.3**	**30.7 ± 5.7**	**3.3 ± 0.8**
N	5	5	5	5	5	5	5	5
P vs $Dd2^{Dd2}$	–	0.310	0.008	0.222	0.222	0.008	0.008	0.151
P vs $Dd2^{GB4}$	0.008	–	–	0.008	0.008	0.008	–	–
P vs $Dd2^{China\ C}$	–	–	–	0.095	0.032		–	–
**25 nM**	**6.9 ± 2.5**	**26.0 ± 5.8**	**5.4 ± 2.3**	**25.9 ± 7.2**	**25.4 ± 7.6**	**40.1 ± 4.5**	**52.8 ± 4.6**	**13.8 ± 2.3**
N	5	5	5	5	5	5	5	5
P vs $Dd2^{Dd2}$	–	0.016	0.691	0.016	0.095	0.008	0.008	0.095
P vs $Dd2^{GB4}$	0.691	–	–	0.016	0.032	0.008	–	–
P vs $Dd2^{China\ C}$	–	–	–	0.222	0.310		–	–
**12.5 nM**	**34.6 ±5.8**	**61.8 ± 4.0**	**42.3 ± 7.5**	**59.9 ± 4.4**	**59 ± 7.9**	**67.0 ± 3.5**	**74.5 ± 3.7**	**46.7 ± 2.7**
N	5	5	5	5	5	5	5	5
P vs $Dd2^{Dd2}$	–	0.032	0.421	0.056	0.056	0.016	0.008	0.151
P vs $Dd2^{GB4}$	0.421	–	–	0.056	0.151	0.016	–	–
P vs $Dd2^{China\ C}$	–	–	–	0.222	0.548	–	–	–
**6.25 nM**	**57.8 ± 3.7**	**76.8 ± 3.1**	**67.4 ± 5.4**	**79.3 ± 3.4**	**86.7 ± 8.7**	**82.2 ± 1.7**	**87.2 ± 3.3**	**72.7 ± 3.0**
N	5	5	5	5	5	5	5	5
P vs $Dd2^{Dd2}$	–	0.008	0.151	0.008	0.056	0.008	0.008	0.016
P vs $Dd2^{GB4}$	0.151	–	–	0.095	0.151	0.016	–	–
P vs $Dd2^{China\ C}$	–	–	–	0.421	0.548	–	–	–
**3.125 nM**	**76.3 ± 2.5**	**87.7 ± 1.9**	**82.5 ± 2.3**	**89.1 ± 2.3**	**93.7 ± 6.9**	**93.7 ± 6.9**	**91.6 ± 1.5**	**86.3 ± 2.3**
N	5	5	5	5	5	5	5	5
P vs $Dd2^{Dd2}$	–	0.016	0.151	0.008	0.056	0.008	0.008	0.032
P vs $Dd2^{GB4}$	0.151	–	–	0.151	0.310	0.008	–	–
P vs $Dd2^{China\ C}$	–	–	–	0.151	0.841	–	–	–

Piperaquine survival assay (PSA) values (nM) indicate the mean ± SEM, as determined in 5 to 6 independent assays performed in duplicate. Parasite survival is defined as the ratio of the parasitemias of the PPQ-treated to the no-drug control wells. This assay measures the survival of synchronous ring-stage parasites (0-6 hr post-invasion) exposed to PPQ for 48 hr and then cultured another 24 hr, prior to measuring parasitemias by flow cytometry. N, number of independent assays. Statistical significance was determined via two-tailed, Student's *t* test. *P* values are reported for comparisons with the parasite lines $Dd2^{Dd2}$, $Dd2^{GB4}$, or $Dd2^{China\ C}$.

*P<0.05	**P<0.01
