# Supplementary material for: Evidence for the early emergence of piperaquine-resistant Plasmodium falciparum malaria and modeling strategies to mitigate resistance
Source: PLoS Pathog. 2022 Feb 7;18(2):e1010278. doi: 10.1371/journal.ppat.1010278 (PMC8853508; doi:10.1371/journal.ppat.1010278)
Supplement: S2 Table — (PDF) [file ppat.1010278.s009.pdf]

S2 Table (page 1 of 2). Antimalarial IC_{50} and IC_{90} values of *pfcrt*-modified parasite lines.

	$Dd2^{Dd2}$	$Dd2^{3D7}$	$Dd2^{GB4}$	$Dd2^{China\ E}$	$Dd2^{China\ B}$	$Dd2^{China\ C}$	$Dd2^{Dd2+F145I}$	$Dd2^{Dd2+A144Y}$
**PPQ IC_{50}**	**13.6 ± 1.5**	**17.1 ± 1.3**	**14.3 ± 1.0**	**20.6 ± 2.9**	**20.6 ± 3.1**	**21.7 ± 2.7**	**27.0 ± 3.2**	**16.3 ± 1.9**
N	6	6	6	6	6	6	6	6
P vs $Dd2^{Dd2}$	–	0.310	0.699	0.132	0.180	0.026	0.004	0.310
P vs $Dd2^{GB4}$	0.6991	–	–	0.2043	0.132	0.0411	–	–
P vs $Dd2^{China\ C}$	–	–	–	0.2403	0.8182	–	–	–
**PPQ IC_{90}**	**28.2 ± 3.6**	**29.4 ± 3.6**	**25.5 ± 1.8**	**36.5 ± 4.3**	**35.4 ± 5.3**	**46.1 ± 2.4**	**n.d.**	**32.6 ± 4.5**
P vs $Dd2^{Dd2}$	–	0.699	0.699	0.132	0.310	0.009	–	0.485
P vs $Dd2^{GB4}$	0.699	–	–	0.041	0.240	0.004	–	–
P vs $Dd2^{China\ C}$	–	–	–	0.247	0.329	–	–	–
**CQ IC_{50}**	**84.5 ± 5.8**	**17.1 ± 2.0**	**56.4 ± 6.0**	**28.7 ± 2.6**	**18.8 ± 1.9**	**11.9 ± 0.9**	**32.0 ± 4.0**	**64.3 ± 7.6**
N	6	6	6	6	5	4	6	4
P vs $Dd2^{Dd2}$	–	0.002	0.026	0.002	0.004	0.010	0.002	0.114
P vs $Dd2^{GB4}$	0.026	–	–	0.002	0.004	0.010	–	–
P vs $Dd2^{China\ B}$	–	–	–	–	–	0.016	–	–
**CQ IC_{90}**	**177.8 ± 16.8**	**25.8 ± 2.4**	**121.5± 15.5**	**53.3 ± 4.7**	**30.1 ± 3.9**	**29.2 ± 1.6**	**75.7 ± 11.6**	**110.9 ± 7.8**
P vs $Dd2^{Dd2}$	–	0.002	0.026	0.002	0.002	0.010	0.002	0.010
P vs $Dd2^{GB4}$	0.026	–	–	0.002	0.002	0.010	–	–
P vs $Dd2^{China\ B}$	–	–	–	–	–	0.476	–	–
**md-CQ IC_{50}**	**437.2 ± 28.9**	**22.4 ± 2.0**	**233.1± 25.1**	**96.0 ± 8.1**	**31.6 ± 2.6**	**22.5 ± 1.2**	**125.6 ± 7.3**	**400.9 ± 14.6**
N	6	5	6	4	5	5	6	4
P vs $Dd2^{Dd2}$	–	0.004	0.002	0.010	0.004	0.010	0.002	0.352
P vs $Dd2^{GB4}$	0.002	–	–	0.010	0.004	0.010	–	–
P vs $Dd2^{China\ B}$	–	–	–	–	–	0.032	–	–
**md-CQ IC_{90}**	**978.7± 54.7**	**42.6 ± 5.7**	**477 ± 21.1**	**201.0 ± 27.6**	**64.6 ± 4.8**	**49.1 ± 3.4**	**299.8 ± 29.7**	**836.0 ± 19.7**
P vs $Dd2^{Dd2}$	–	0.004	0.002	0.010	0.004	0.004	0.002	0.010
P vs $Dd2^{GB4}$	0.002	–	–	0.010	0.004	0.004	–	–
P vs $Dd2^{China\ B}$	–	–	–	–	–	0.008	–	–
**md-ADQ IC_{50}**	36.5 ± 2.2	15.8 ± 1.8	18.8 ± 1.2	20.76 ± 3.8	24.0 ± 3.4	18.1 ± 2.1	24.2 ± 3.0	42.1 ± 5.7
N	7	7	7	6	5	5	7	5
P vs $Dd2^{Dd2}$	–	0.001	0.001	0.022	0.005	0.003	0.011	0.202
P vs $Dd2^{GB4}$	0.001	–	–	>0.999	0.149	>0.999	–	–
P vs $Dd2^{China\ B}$	–	–	–	–	–	0.222	–	–
**md-ADQ IC_{90}**	**59.9 ± 0.8**	**23.8 ± 2.3**	**28.5 ± 0.5**	**30.7 ± 5.6**	**44.1 ± 7.5**	**31.0 ± 4.0**	**38.5 ± 4.4**	**67.2 ± 10.1**
P vs $Dd2^{Dd2}$	–	0.001	0.001	0.022	0.010	0.003	0.011	0.202
P vs $Dd2^{GB4}$	0.001	–	–	>0.999	0.106	0.432	–	–
P vs $Dd2^{China\ B}$	–	–	–	–	–	0.222	–	–
**QN IC_{50}**	151.7 ± 17.0	227.5 ± 26.6	175.7 ± 22.8	221.5 ± 21.8	123.0 ± 15.6	116.2± 10.5	130.4 ± 10.1	176.5 ± 13.8
N	6	6	6	6	5	6	6	4
P vs $Dd2^{Dd2}$	–	0.041	0.589	0.041	0.662	0.310	0.558	0.476
P vs $Dd2^{GB4}$	0.589	–	–	0.132	0.178	0.065	–	–
P vs $Dd2^{China\ B}$	–	–	–	–	–	0.792	–	–
**QN IC_{90}**	**557.8 ± 38.6**	**476.5 ± 53.6**	**567.5 ± 46.1**	**694.1 ± 50.4**	**448.6 ± 16.3**	**451.1 ± 34.2**	**523.7 ± 32.8**	**501.9 ± 20.8**
P vs $Dd2^{Dd2}$	–	0.310	>0.9999	0.052	0.065	0.310	0.457	0.281
P vs $Dd2^{GB4}$	0.589	–	–	0.093	0.052	0.093	–	–
P vs $Dd2^{China\ B}$	–	–	–	–	–	>0.9999	–	–
**PND IC_{50}**	**7.6 ± 0.6**	**7.0 ± 0.7**	**8.3 ± 0.5**	**7.7 ± 1.3**	**6.9 ± 1.3**	**9.7 ± 0.5**	**8.1± 1.2**	**10.2 ± 0.9**
N	6	6	6	5	4	5	6	5
P vs $Dd2^{Dd2}$	–	0.589	0.485	0.931	0.857	0.052	0.818	0.030
P vs $Dd2^{GB4}$	0.589	–	–	0.792	0.438	0.126	–	–
P vs $Dd2^{China\ B}$	–	–	–	–	–	0.064	–	–
**PND IC_{90}**	**11.6 ± 0.2**	**10.6 ± 0.9**	**12.2 ± 0.3**	**14.7 ± 2.5**	**13.6 ± 2.7**	**19.7 ± 1.4**	**15.4 ± 2.4**	**18.3 ± 1.7**
P vs $Dd2^{Dd2}$	–	0.420	0.240	0.931	0.762	0.004	0.699	0.004
P vs $Dd2^{GB4}$	0.420	–	–	0.662	>0.9999	0.004	–	–
P vs $Dd2^{China\ B}$	–	–	–	–	–	0.064	–	–

S2 Table (page 2 of 2). Antimalarial IC_{50} and IC_{90} values of *pfcrt*-modified parasite lines.

	**$Dd2^{Dd2}$**	**$Dd2^{GCO3}$**	**$Dd2^{GB4}$**	**$Dd2^{China\ E}$**	**$Dd2^{China\ B}$**	**$Dd2^{China\ C}$**	**$Dd2^{Dd2+F145I}$**	**$Dd2^{Dd2+A144Y}$**
**FQ IC_{50}**	**8.2 ± 0.5**	**5.5 ± 0.5**	**7.1 ± 0.6**	**8.3 ± 1.0**	**7.8 ± 0.7**	**8.6 ± 0.3**	**8.1 ± 01.0**	**8.3 ± 0.5**
N	8	8	8	8	7	6	6	8
P vs $Dd2^{Dd2}$	–	0.002	0.205	0.798	0.717	0.687	0.879	>0.9999
P vs $Dd2^{GB4}$	0.205	–	–	0.279	0.536	0.108	–	–
P vs $Dd2^{China\ B}$	–	–	–	–	–	0.445	–	–
**FQ IC_{90}**	**11.2 ± 0.4**	**8.7 ± 0.9**	**10.8 ± 0.7**	**15.2 ± 4.4**	**11.7 ± 1.6**	**13.23 ± 1.5**	**12.5± 2.1**	**11.4 ± 0.3**
P vs $Dd2^{Dd2}$	–	0.021	0.985	0.958	0.845	0.573	0.852	0.859
P vs $Dd2^{GB4}$	0.985	–	–	0.742	0.807	0.573	–	–
P vs $Dd2^{China\ B}$	–	–	–	–	–	0.295	–	–
**DHA IC_{50}**	**2.1± 0.2**	**2.2 ± 0.3**	**2.0 ± 0.3**	**2.1 ± 0.2**	**2.2 ± 0.2**	**1.8 ± 0.3**	**1.9 ± 0.4**	**2.0 ± 0.2**
N	5	4	5	5	5	4	4	5
P vs $Dd2^{Dd2}$	–	0.730	>0.999	0.841	0.841	0.413	0.556	0.841
P vs $Dd2^{GB4}$	>0.999	–	–	>0.999	0.694	0.730	–	–
P vs $Dd2^{China\ B}$	–	–	–	–	–	0.413	–	–
**DHA IC_{90}**	**4.9 ± 0.2**	**4.9 ± 0.7**	**3.9 ± 0.6**	**4.6 ± 0.5**	**4.9 ± 0.3**	**4.8 ± 1.2**	**4.4 ± 0.6**	**4.4 ± 0.6**
P vs $Dd2^{Dd2}$	–	>0.999	0.310	0.841	0.717	0.687	0.286	>0.9999
P vs $Dd2^{GB4}$	0.310	–	–	0.310	>0.999	>0.999	–	–
P vs $Dd2^{China\ B}$	–	–	–	–	–	>0.999	–	–
**LMF IC_{50}**	**1.2 ± 0.1**	**2.1 ± 0.2**	**1.2 ± 0.2**	**1.1 ± 0.1**	**1.3 ± 0.2**	**1.4 ± 0.2**	**1.5 ± 0.2**	**1.3 ± 0.1**
N	6	5	7	6	5	6	6	7
P vs $Dd2^{Dd2}$	–	0.004	0.836	0.853	0.931	0.589	0.485	0.761
P vs $Dd2^{GB4}$	0.836	–	–	0.918	0.530	0.249	–	–
P vs $Dd2^{China\ B}$	–	–	–	–	–	0.697	–	–
**LMF IC_{90}**	**7.6 ± 1.5**	**6.5 ± 0.6**	**8.8 ± 1.3**	**7.8 ± 1.0**	**5.8 ± 0.6**	**8.2 ± 1.5**	**6.2± 0.8**	**5.8 ± 0.6**
P vs $Dd2^{Dd2}$	–	0.792	0.628	0.818	0.429	>0.9999	0.589	0.295
P vs $Dd2^{GB4}$	0.628	–	–	0.534	0.149	0.731	–	–
P vs $Dd2^{China\ B}$	–	–	–	–	–	0.429	–	–
**MFQ IC_{50}**	**6.4 ± 07**	**11.54 ± 1.0**	**6.6 ± 0.7**	**7.5 ± 1.1**	**6.6 ± 0.7**	**5.7 ± 0.3**	**7.7 ± 0.7**	**5.5 ± 0.6**
N	7	6	7	7	7	6	5	7
P vs $Dd2^{Dd2}$	–	0.002	0.902	0.456	0.805	0.731	0.343	0.209
P vs $Dd2^{GB4}$	0.902	–	–	0.710	0.902	0.234	–	–
P vs $Dd2^{China\ B}$	–	–	–	–	–	0.445	–	–
**MFQ IC_{90}**	**20.3 ± 1.8**	**28.6 ± 2.8**	**22.1 ± 2.4**	**2.1 ± 2.0**	**20.4 ± 1.6**	**19.2 ± 1.1**	**20.9 ± 1.5**	**18.1 ± 1.2**
P vs $Dd2^{Dd2}$	–	0.022	0.710	0.456	0.902	0.445	>0.999	0.259
P vs $Dd2^{GB4}$	0.710	–	–	0.805	0.620	0.295	–	–
P vs $Dd2^{China\ B}$	–	–	–	–	–	0.295	–	–

IC_{50} and IC_{90} values (nM) are presented as the means ± SEM, as determined by regression analysis in 4 to 8 independent assays perfomed in duplicate. PPQ, piperaquine; CQ, chloroquine; md-CQ, monodesethyl-chloroquine; md-ADQ, monodesethyl-amodiaquine; QN, quinine; PND, pyronaridine; FQ, ferroquine; DHA, dihydroartemisinin; LMF, lumefantrine; MFQ, mefloquine; N, number of assays; n.d., not determined. Statistical significance was determined via two-tailed Mann Whitney *U* tests. *P* values are reported for comparisons with the parasite line $Dd2^{Dd2}$.

**P*<0.05	***P*<0.01	***P<0.001
