# Supplementary material for: Evidence for the early emergence of piperaquine-resistant Plasmodium falciparum malaria and modeling strategies to mitigate resistance
Source: PLoS Pathog. 2022 Feb 7;18(2):e1010278. doi: 10.1371/journal.ppat.1010278 (PMC8853508; doi:10.1371/journal.ppat.1010278)
Supplement: S3 Table — (PDF) [file ppat.1010278.s010.pdf]

S3 Table. CQ and PPQ transport by PfCRT-containing proteoliposomes.

	CQ			PPQ		
PfCRT Isoform	1-min uptake (nmol x mg^{-1})	K_m (µM)	V_{max} (nmol x mg^{-1} x min-^{1})	1-min uptake (nmol x mg^{-1})	K_m (µM)	V_{max} (nmol x mg^{-1} x min^{-1})
China C	2.11 ± 0.03	0.17 ± 0.02	34.7 ± 1.2	4.44 ± 0.19	0.15 ± 0.04	100.0 ± 7.1
Dd2+F145I	2.83 ± 0.19	0.17 ± 0.04	50.1 ± 2.9	4.32 ± 0.38	0.38 ± 0.13	142.7 ± 15.6
Dd2	3.53 ± 0.16	0.48 ± 0.06	89.1 ± 3.6	0.52 ± 0.18	n.d.	n.d.
3D7	0.77 ± 0.26	n.d.	n.d.	1.56 ± 1.04	n.d.	n.d.
Control	0.71 ± 0.24	n.d.	n.d.	1.21 ± 0.65	n.d.	n.d.

Transport measurements for ^{3}H-CQ and ^{3}H-PPQ uptake were determined with PfCRT variants reconstituted into proteoliposomes or with empty liposomes (control) using an inwardly-directed pH gradient and membrane potential (inside negative). The mean±SEM for levels of uptake after 1 min with ^{3}H-CQ or ^{3}H-PPQ (depicted in **Fig. 2A-B**) are shown for each variant tested (N = 4 independent experiments). The concentration-dependent uptake of ^{3}H-CQ or ^{3}H-PPQ (shown as mean±SEM) shown in **Fig. 2C-D** were fitted to the Michaelis–Menten equation in GraphPad Prism to obtain K_m and V_{max} values (N = 3-4 independent experiments). n.d., not determined because of low transport activities.
