# Supplementary material for: Evidence for the early emergence of piperaquine-resistant Plasmodium falciparum malaria and modeling strategies to mitigate resistance
Source: PLoS Pathog. 2022 Feb 7;18(2):e1010278. doi: 10.1371/journal.ppat.1010278 (PMC8853508; doi:10.1371/journal.ppat.1010278)
Supplement: S4 Table — (PDF) [file ppat.1010278.s011.pdf]

S4 Table. Normalized dose response curves for piperaquine and chloroquine.

	Piperaquine			Chloroquine		
Line	IC_{50} (nM)	Hill Slope	S	IC_{50} (nM)	Hill Slope	S
$Dd2^{Dd2}$	9.1	-1.24	2.36E+10	82.0	-3.57	0.92
$Dd2^{Dd2+T356I}$	12.4	-2.89	2.19	23.4	-3.16	1.38
$Dd2^{Dd2+S326N}$	13.5	-2.68	3.05	25.4	-8.57	0.38
$Dd2^{Dd2+A144Y}$	12.1	-1.38	36.88	63.4	-2.62	3.62
$Dd2^{Dd2+F145I*}$	28.1	-1.34	1.04	27.0	-1.97	3.73
$Dd2^{Dd2+T93S}$	28.0	-3.69	0.31	73.6	-1.99	2.68E+12
$Dd2^{Dd2+I218F}$	26.7	-3.86	0.26	42.7	-2.96	0.61
$Dd2^{GB4}$	11.2	-1.30	1.62E+12	54.5	-4.17	0.60
$Dd2^{China\ E}$	16.4	-1.41	9.14E+13	28.8	-4.80	0.63
$Dd2^{China\ C}$	19.2	-1.75	1.10	12.2	-6.99	0.27
$Dd2^{China\ B}$	16.0	-1.71	4.28	17.5	-3.79	1.30

Curves were fit using an asymmetric 5-parameter Richards equation using Prism, with bottom and top values constrained to 0 and 1, respectively. The values of the remaining 3 free parameters of the fitted curves for each of the strains are shown above. For each line and drug, the average percent inhibition was calculated from the 4 to 6 independent repeats (with technical duplicates) for each drug concentration, and the Richards equation used to extrapolate a single IC_{50} value. *For concentrations x = 100 or greater, the Dd2+F145I dose response to PPQ was fit with the quadratic polynomial: $y = -0.2295x^2 + 1.3303x - 1.5952$
