# Supplementary material for: Evidence for the early emergence of piperaquine-resistant Plasmodium falciparum malaria and modeling strategies to mitigate resistance
Source: PLoS Pathog. 2022 Feb 7;18(2):e1010278. doi: 10.1371/journal.ppat.1010278 (PMC8853508; doi:10.1371/journal.ppat.1010278)
Supplement: S5 Table — (PDF) [file ppat.1010278.s012.pdf]

S5 Table. Drug concentrations utilized in the simulator.

Drug	Half-life due to absorption $t_{1/2abs}$ (days)	Half-life due to elimination $t_{1/2\beta}$ (days)
PPQ	0.346	17.208
CQ	0.533	9.708

Drug concentrations were assumed to remain constant during the first 3 days of administration given the one day timestep. Concentrations then followed a multi-exponential decay, dropping rapidly on day 4 of treatment due to absorption and then more slowly thereafter due to elimination. The median absorption and elimination half-life measurements were those reported in Karunajeewa *et al.* 2008 Antimicrob Agents Chemother; PMID: 17967917. These values were determined by fitting a 2-compartment model with first-order absorption kinetics to PPQ and CQ data. These half-life values were then converted from hours to days for the simulation.
