# Supplementary material for: Evidence for the early emergence of piperaquine-resistant Plasmodium falciparum malaria and modeling strategies to mitigate resistance
Source: PLoS Pathog. 2022 Feb 7;18(2):e1010278. doi: 10.1371/journal.ppat.1010278 (PMC8853508; doi:10.1371/journal.ppat.1010278)
Supplement: S6 Table — (PDF) [file ppat.1010278.s013.pdf]

S6 Table. Summary of simulation outcomes for single parasite strain infections of $Dd2^{China\ B}$, $Dd2^{ChinaE}$, $Dd2^{Dd2+T356I}$, $Dd2^{Dd2+I218F}$, $Dd2^{Dd2+A144Y}$, $Dd2^{Dd2+S326N}$, and $Dd2^{GB4}$ with initial treatment with piperaquine.

Initial Piperaquine Concentration (nM)	Optional Chloroquine Concentration (nM)	China B	China E	Dd2+T356I	Dd2+I218F**	Dd2+A144Y	Dd2+S326N	GB4
Low PPQ: 200	No CQ	None (100%)	None (100%)	None (100%)	Dd2+I218F (100%)	None (100%)	None (100%)	None (100%)
	Late Rescue CQ: 125	None* (100%)	None* (100%)	None* (100%)	Dd2+I218F (100%)	None* (100%)	None* (100%)	None* (100%)
	Sequential CQ: 125	None* (100%)	None* (100%)	None* (100%)	Dd2+I218F (100%)	None* (100%)	None* (100%)	None* (100%)
	Simultaneous CQ: 125	None (100%)	None (100%)	None (100%)	Dd2+I218F (100%)	None (100%)	None (100%)	None (100%)
High PPQ: 400	No CQ	None (100%)	None (100%)	None (100%)	Dd2+I218F (100%)	None (100%)	None (100%)	None (100%)
	Late Rescue CQ: 250	None* (100%)	None* (100%)	None* (100%)	Dd2+I218F (100%)	None* (100%)	None* (100%)	None* (100%)
	Sequential CQ: 250	None* (100%)	None* (100%)	None* (100%)	None (100%)	None* (100%)	None* (100%)	None* (100%)
	Simultaneous CQ: 250	None (100%)	None (100%)	None (100%)	None (100%)	None (100%)	None (100%)	None (100%)

Data are based on isogenic parasite lines ($Dd2^{China\ B}$, $Dd2^{China\ E}$, $Dd2^{Dd2+T356I}$, $Dd2^{Dd2+I218F}$, $Dd2^{Dd2+A144Y}$, $Dd2^{Dd2+S326N}$, and $Dd2^{GB4}$) differing only in their PfCRT isoform as noted above. Simulations were performed with parasites exposed to 8 possible treatment regimens, starting with treatment by piperaquine (PPQ) with optional treatment with chloroquine (CQ). "Late Rescue" was triggered by the simulation when parasites recrudesced to the treatment threshold; "Sequential" means that CQ was dosed immediately after the PPQ dose completed (i.e. on day 4); "Simultaneous CQ" means that PPQ and CQ were administered at the same time. Entries indicate the most dominant strains at the ends of 100 stochastic simulations of each treatment regimen. **None** indicates that simulated treatment was successful in killing all the parasites. **None*** means that the scheduled rescue did not occur because all the parasites died before rescue was triggered.

**$Dd2^{Dd2+I218F}$ was included here as the only PPQ-resistant line.
