# Supplementary material for: Evidence for the early emergence of piperaquine-resistant Plasmodium falciparum malaria and modeling strategies to mitigate resistance
Source: PLoS Pathog. 2022 Feb 7;18(2):e1010278. doi: 10.1371/journal.ppat.1010278 (PMC8853508; doi:10.1371/journal.ppat.1010278)
Supplement: S7 Table — (PDF) [file ppat.1010278.s014.pdf]

**S7 Table.** Summary of simulation outcomes for single parasite strain infections of $Dd2^{Dd2}$, $Dd2^{Dd2+F145I}$, $Dd2^{Dd2+T93S}$, $Dd2^{China\ C}$ with initial treatment with chloroquine.

| Initial Chloroquine (CQ) Concentration (nM) | Optional Piperaquine (PPQ) Concentration (nM) | Dd2 | Dd2+F145I | Dd2+T93S | China C |
|---|---|---|---|---|---|
| Low CQ: 125 | No PPQ | Dd2 (100%) | Dd2 (100%) | Dd2+T93S (100%) | None (83%)<br>HI2 (16%)<br>China C (1%) |
| | Late Rescue PPQ: 200 | Dd2+T93S (100%) | Dd2+T93S (100%) | Dd2+T93S (100%) | None* (83%)<br>HI2 (16%)<br>China C (1%) |
| | Sequential Rescue PPQ: 200 | None (78%)<br>Dd2+T93S (17%)<br>Dd2+I218F (4%)<br>Dd2+F145I (1%) | Dd2+F145I (85%)<br>None (15%) | Dd2+T93S (100%) | None (100%) |
| High CQ: 250 | No PPQ | Dd2 (100%) | Dd2 (100%) | Dd2+T93S (100%) | None (83%) |
| | Late Rescue PPQ: 400 | None (99%)<br>Dd2+T93S (1%) | None (89%)<br>Dd2+F145I (10%)<br>Dd2+T93S (1%) | Dd2+T93S (100%) | None* (100%) |
| | Sequential Rescue PPQ: 200 | None (100% | None (100%) | None (100%) | None (100%) |

Data are based on isogenic parasite lines ($Dd2^{Dd2}$, $Dd2^{Dd2+F145I}$, $Dd2^{Dd2+T93S}$, and $Dd2^{China\ C}$) differing only in their *pfcrt* allele as noted above. Simulations were performed with parasites exposed to 6 possible treatment regimens, starting with treatment by chloroquine (CQ) with optional "Late Rescue" treatment with piperaquine (PPQ), which was triggered by the simulation when parasites recrudesced to the treatment threshold. "Sequential" means that PPQ was dosed immediately after the CQ dose completed (i.e. on day 4). Entries indicate the most dominant strains at the ends of 100 stochastic simulations of each treatment regimen. **None** indicates that simulated treatment was successful in killing all the parasites. **None*** means that the scheduled rescue did not occur because all the parasites died before rescue was triggered. HI2, hypothetical intermediate 2.
