# Supplementary material for: Evidence for the early emergence of piperaquine-resistant Plasmodium falciparum malaria and modeling strategies to mitigate resistance
Source: PLoS Pathog. 2022 Feb 7;18(2):e1010278. doi: 10.1371/journal.ppat.1010278 (PMC8853508; doi:10.1371/journal.ppat.1010278)
Supplement: S8 Table — (PDF) [file ppat.1010278.s015.pdf]

S8 Table. Summary of simulation outcomes for single parasite strain infections of Dd2$^{China\ B}$, Dd2^{ChinaE}, Dd2$^{Dd2+T356I}$, Dd2$^{Dd2+I218F}$, Dd2$^{Dd2+A144Y}$, Dd2$^{Dd2+S326N}$, and Dd2^{GB4} with initial treatment with chloroquine.

Initial Chloroquine (CQ) Concentration (nM)	Optional Piperaquine (PPQ) Concentration (nM)	China B	China E	Dd2+T356I	Dd2+I218F	Dd2+A144Y	Dd2+S326N	GB4
Low CQ: 125	No PPQ	None (100%)	China E (100%)	Dd2 (100%)	Dd2+I218F (100%)	Dd2+A144Y (100%)	Dd2+S326N (70%)<br>Dd2 (30%)	GB4 (100%)
	Late Rescue PPQ: 200	None* (100%)	None (100%)	Dd2+T93S (100%)	Dd2+I218F (100%)	None (100%)	Dd2+T93S (98%)<br>Dd2+I218F (2%)	None (100%)
	Sequential Rescue PPQ: 200	None (100%)	None (100%)	None (100%)	Dd2+I218F (100%)	None (100%)	None (100%)	None (100%)
High CQ: 250	No PPQ	None (100%)	None (96%)<br>GB4 (4%)	None (56%)<br>Dd2 (44%)	Dd2+I218F (100%)	Dd2 (60%)<br>Dd2+F145I (40%)	None (82%)<br>Dd2 (18%)	GB4 (100%)
	Late Rescue PPQ: 400	None* (100%)	None* (96%)<br>None (4%)	None* (56%)<br>None (38%)<br>Dd2+F145I (5%)<br>Dd2+I218F (1%)	Dd2+I218F (100%)	None (91%)<br>Dd2+F145I (9%)	None* (82%)<br>None (12%)<br>Dd2+F145I (5%)<br>Dd2+T93S (1%)	None (100%)
	Sequential Rescue PPQ: 400	None* (100%)	None (100%)	None (58%)<br>None* (42%)	None (100%)	None (100%)	None (100%)	None (100%)

Data are based on isogenic parasite lines (Dd2$^{China\ B}$, Dd2^{ChinaE}, Dd2$^{Dd2+T356I}$, Dd2$^{Dd2+I218F}$, Dd2$^{Dd2+A144Y}$, Dd2$^{Dd2+S326N}$, and Dd2^{GB4}) differing only in their *pfcrt* allele as noted above. Simulations were performed with parasites exposed to 6 possible treatment regimens, starting with treatment by chloroquine (CQ) with optional "Late Rescue" treatment with piperaquine (PPQ), which was triggered by the simulation when parasites recrudesced to the treatment threshold. "Sequential" means that PPQ was dosed immediately after the CQ dose completed (i.e. on day 4). Entries indicate the most dominant strains at the end of 100 stochastic simulations of each treatment regimen. **None** indicates that simulated treatment was successful in killing all the parasites. **None*** means that the scheduled rescue did not occur because all the parasites died before rescue was triggered.
