# Supplementary material for: Evidence for the early emergence of piperaquine-resistant Plasmodium falciparum malaria and modeling strategies to mitigate resistance
Source: PLoS Pathog. 2022 Feb 7;18(2):e1010278. doi: 10.1371/journal.ppat.1010278 (PMC8853508; doi:10.1371/journal.ppat.1010278)
Supplement: S9 Table — (PDF) [file ppat.1010278.s016.pdf]

S9 Table. List of oligonucleotides used in this study

Name	Nucleotide Sequence (5'-3')	Description	Lab name	Purpose
p1	CCCTTGTCGACCTTAACAGATGGCTC	*pfcrt* exon 2 SalI Forward	p3519	Sequencing primer for *pfcrt*
p2	TCAAACATGACAAGGGAAATAGT	*pfcrt* exon 5 Reverse	p2427	
p3	CTCGAGatggttggttcgctaaactgc	*hDHFR* XhoI forward	p3315	Integration PCR #1. 2.5 kb yes/no integration at 3' end Sequences exons 2-3
p4	TTGACCCTTATATATTCCACCCA	*pfcrt* 3' UTR (+1336)	p3403	
p5	cttgggCCCAAGTTGTACTGCTTCTAAGC	*pfcrt* 5' UTR (-494-517) ApaI Forward	p3404	Integration PCR #2. 1.2 kb checks integration at 5' end (1.4 kb if unedited due to additional intron 2)
p6	cttatcgatAAGCAGAAGAACATATTAATAG GAATACTTAATTG	*pfcrt* exon 3 ClaI Reverse	p3265	
p7	GACCTTAACAGATGGCTCAC	*pfcrt* exon 2 EcoRI Forward	p3264	Integration PCR #3 primer (along with p6). 0.4 kb (0.6 kb if unedited due to additional intron 2). Sequences exons 2-3
p8	aaccatggatTTATTGTGTAATAATTGAATCG ACG	*pfcrt* exon 13 Reverse	p1640	Integration PCR #4 primer (along with p5). 2 kb. Sequence entire edited locus
p9	agccGGTGATGTTGTAAgAGAACCAAGATT ATTAG	PfCRT I317R SDM Forward	p7068	PfCRT I317R SDM
p10	CTAATAATCTTGGTTCTcTTACAACATCAC Cggct	PfCRT I317R SDM Reverse	p7069	
p11	CTGTTCAGTCATTTTGtaCTTCATAGGTCT TACAA	PfCRT A144Y SDM Forward	p7070	PfCRT A144Y SDM
p12	TTGTAAGACCTATGAAGtaCAAAATGACTG AACAG	PfCRT A144Y SDM Reverse	p7071	
p13	cttgtcttaattagtGccttaattCCTGTATGC	PfCRT S220A SDM Forward	p7072	PfCRT S220A SDM
p14	GCATACAGGaattaaggCactaattaagacaag	PfCRT S220A SDM Reverse	p7073	
p15	tattatttatttaagtgtatgtgtaatTGaCaCaatttttgctaaa agaactttaaac	PfCRT E75D SDM Forward	p7430	PfCRT E75D SDM
p16	gtttaaagttcttttagcaaaaattGtGtCAattacacataca cttaaataaataata	PfCRT E75D SDM Reverse	p7431	
p17	agccGGTGATGTTGTAAtAGAACCAAGATT ATTAG	PfCRT R371I SDM Forward	p7076	PfCRT R371D SDM
p18	CTAATAATCTTGGTTCTaTTACAACATCAC Cggct	PfCRT R371I SDM Reverse	p7077	
p19	TCGGTAATCCTGGCAaTCATTGGCCTCA CTCGT	PfCRT F145I SDM Forward	p7635	PfCRT F145I SDM on *S. cerevisiae* codon optimized
p20	ACGAGTGAGGCCAATGAtTGCCAGGATTA CCGA	PfCRT F145I SDM Forward	p7636	
p21	CCGCGACTAGTGAGCTCGTCGAC	pFastBac Sequencing Reverse	p8157	Sequencing primer for pFastBac constructs
