# Supplementary material for: Evidence for the early emergence of piperaquine-resistant Plasmodium falciparum malaria and modeling strategies to mitigate resistance
Source: PLoS Pathog. 2022 Feb 7;18(2):e1010278. doi: 10.1371/journal.ppat.1010278 (PMC8853508; doi:10.1371/journal.ppat.1010278)
Supplement: S1 Text — (PDF) [file ppat.1010278.s017.pdf]

Supplementary Materials and Methods

Plasmid Construction, Parasite Culturing and Transfections. *pfcrt* was edited using customized zinc-finger nucleases and a two-plasmid approach that replaces the endogenous allele with a recombinant allele containing the mutations of interest [1]. Mutations were introduced into a *pfcrt*^{GB4} donor plasmid by site-directed mutagenesis (**S1B Fig; S9 Table**). Dd2 *P. falciparum* ABS parasites were cultured and transfected as described in [2]. The donor plasmid carrying the human *dhfr* marker was selected with 2.5 nM WR99210 (Jacobus Pharmaceuticals), and the zinc-finger nuclease plasmid harboring blasticidin S-deaminase was selected with a 6-day pulse of 2 µg/ml blasticidin hydrochloride (ThermoFisher). Editing was confirmed using PCR primers p1-7 (**S1B Fig; S9 Table**) and Sanger sequencing, and clones were obtained by limiting dilution.

Piperaquine Survival Assays. For PSAs, we seeded tightly sorbitol-synchronized ring stage parasites (0-6 hr post invasion) at 1% parasitemia and 1% hematocrit in 96-well flat-bottom plates containing 10 point, 2-fold dilutions of PPQ with a maximum of 1600 nM [3-5]. Parasites were incubated for 48 hr at 37°C, washed three times with complete media to remove drug (using a TECAN Freedom EVO 100 liquid handler), then cultured an additional 24 hr. Parasites were labeled with SYBR Green I and MitoTracker Deep Red (as DNA and mitochondrial dyes, respectively) and parasitemias measured on an iQue Plus flow cytometer. Percent survival was calculated by dividing the parasitemia of the PPQ-treated parasites by that of the no-drug control [4,5]. Statistical significance was determined using non-parametric, two-tailed Mann-Whitney *U* tests (GraphPad Prism 8 software). Raw data and statistics are listed in **S1 Table**.

Drug Susceptibility Assays. Asynchronous, ABS parasites were plated at 0.3-0.5% parasitemia and 1% hematocrit in 96-well plates, and incubated with a 10 point, 2-fold range of drug concentrations. Plates were incubated at 37°C for 72 hr, and parasitemias were measured by flow cytometry. IC_{50} values were calculated by nonlinear regression analysis. Statistical significance was determined using Mann-Whitney *U* tests (**S2 Table**).

PfCRT Protein Expression and Purification. The *pfcrt* 7G8, HB3, Dd2, Dd2+F145I, and China C full-length open-reading frames were cloned into the pEG BacMam vector [6] using InFusion cloning. *pfcrt* was fused at its 3′ end with a Tobacco Etch Virus protease cleavage site (ENLYFQSYV) and a deca-histidine affinity tag followed by a streptavidin affinity tag (WSHPQFEK). Bacmids and recombinant P1 baculovirus were prepared as described in [7]. To prepare recombinant protein, we infected HEK293S GnTi-negative cells (Invitrogen) with P4 virus and used Ni^{2+}-NTA resin chromatography to purify PfCRT from n-dodecyl-β-d-maltopyranoside (DDM) and cholesteryl hemisuccinate (CHS)-solubilized membrane extracts [7,8].

Kinetic Transport Constant Calculations. K_m and V_{max} were determined by measuring the rates of transport for different concentrations of ^{3}H-CQ or ^{3}H-PPQ at 10 seconds and fitting the data to the Michaelis-Menten equation (GraphPad Prism 8; values shown in **S3 Table**).

Molecular Dynamic Simulations. Calculations were performed using the Schrödinger molecular modelling suite (version 2019-1) and variant PfCRT protein structures modeled using the Protein Preparation Wizard [9]. In this step, force field atom types and bond orders were assigned, missing atoms including hydrogens were added, tautomer/ionization states were assigned, Asn, Gln and His residues were flipped to optimize the hydrogen bond network, and energy minimization was constrained. To model Dd2, WT (3D7), China C, and Dd2+F145I, mutations were introduced into the PfCRT 7G8 structure using the Residue and Loop Mutation tool. The refinement step consisted of local minimization in implicit solvent with Prime [10]. Molecular dynamics simulations used the same protocol for all isoforms. First, the protein was aligned so that the membrane plane corresponded to the *xy* plane of the coordinate system, using the Orientation of Proteins in Membranes database [11]. Then, the protein was embedded in a 1-palmitoyl-2-oleoyl-glycero-3-phospho-choline (POPC) membrane model, included in an orthorhombic box of simple point-charge (SPC) water solvent with a 10 Å buffer on each dimension, and neutralized by adding the appropriate number of Cl^- ions, using the System Builder panel. Each simulation, performed using Desmond, started with a system relaxation and a default protocol that was followed by a 300-ns production

run in an NPgT ensemble, at a temperature of 300 K and a pressure of 1.01325 bar. Trajectories were analyzed using the Simulation Event Analysis tool to monitor distances of interest, and the frames for each trajectory were clustered using Desmond Trajectory. The Poisson–Boltzmann electrostatic potential surface panel, which uses the adaptive Poisson–Boltzmann solver method, was used to calculate the electrostatic potential surfaces. The solute and solvent dielectric constants were set to 1 and 80, respectively, solvent radius was 1.4 Å, temperature was 298 K and grid extension was 5 Å.

***In vitro* Fitness Assays.** Parasites were co-cultured in a 1:1 ratio with a Dd2-GFP$^+$ reporter parasite line in 96-well plates. Parasites were stained with MitoTracker Deep Red and growth monitored for 26 days on an iQue Plus flow cytometer. Relative growth rates of each test line are shown by plotting the percentage of GFP$^+$ cells in culture over time [4,5].

Parasite Expansion Rate Calculations. We took the expansion rate of each parasite line under no drug and subtracted the background, then divided this by the seeded parasitemia. For the lines profiled herein (Dd2^{Dd2}, Dd2^{3D7}, Dd2$^{Dd2+F145I}$, Dd2$^{Dd2+A144Y}$, Dd2^{GB4}, Dd2$^{China\ E}$, Dd2$^{China\ B}$ and Dd2$^{China\ C}$) the averages were derived from 57 total drug assays run on 15 separate days. For the Dd2$^{Dd2+T93S}$ and Dd2$^{Dd2+I218F}$ lines, the averages were from 58 drug assays over 6 separate days, and for Dd2$^{Dd2+S326N}$ and Dd2$^{Dd2+T356I}$ these were from 16 or 19 drug assays, respectively, conducted over four separate days. The reference Dd2^{Dd2} line was assayed in every experiment. To obtain the 24-hr no-drug expansion rates (**Fig 3B**) we used the equation 10^((log(72-hr expansion rate)/3)). These rates correlated with relative fitness data obtained from competitive growth assays reported herein (**Fig. 3A**) or previously published [5,12].

To estimate growth rates in the presence of variable concentrations of either CQ or PPQ, we used our available dose-response data (**Fig. 1**) [5,12]. Data points were first normalized to the absolute growth rate in the absence of drug (in the range of 0-1 [13], where 0 means that no parasites survived and 1 means that all parasites survived) and then averaged over replicates at each given dose (**S6 Fig**). These averaged measurements were then fitted using the 5-parameter asymmetric Richards equation (GraphPad Prism 8)

with lower and upper bounds constrained to 0 and 1, respectively. The only exception to this was $Dd2^{Dd2+F145I}$ growth under high PPQ concentrations that was fitted with a quadratic polynomial, due to its consistently non-monotonic growth pattern (**Fig. 1**). Fitted parameters and relative growth rate curves are shown in **S4 Table** and **S6 Fig**, respectively.

Supplementary References

1. Straimer J, Lee MC, Lee AH, Zeitler B, Williams AE*, et al.* Site-specific genome editing in *Plasmodium falciparum* using engineered zinc-finger nucleases. Nat Methods. 2012; 9: 993-8. https://doi.org/10.1038/nmeth.2143 PMID: 22922501
2. Fidock DA, Nomura T and Wellems TE. Cycloguanil and its parent compound proguanil demonstrate distinct activities against *Plasmodium falciparum* malaria parasites transformed with human dihydrofolate reductase. Mol Pharmacol. 1998; 54: 1140-7. https://doi.org/10.1124/mol.54.6.1140 PMID: 9855645
3. Witkowski B, Duru V, Khim N, Ross LS, Saintpierre B*, et al.* A surrogate marker of piperaquine-resistant *Plasmodium falciparum* malaria: a phenotype-genotype association study. Lancet Infect Dis. 2017; 17: 174-83. https://doi.org/10.1016/S1473-3099(16)30415-7 PMID: 27818097
4. Ross LS, Dhingra SK, Mok S, Yeo T, Wicht KJ*, et al.* Emerging Southeast Asian PfCRT mutations confer *Plasmodium falciparum* resistance to the first-line antimalarial piperaquine. Nat Commun. 2018; 9: 3314. https://doi.org/10.1038/s41467-018-05652-0 PMID: 30115924
5. Dhingra SK, Small-Saunders JL, Menard D and Fidock DA. *Plasmodium falciparum* resistance to piperaquine driven by PfCRT. Lancet Infect Dis. 2019; 19: 1168-9. https://doi.org/10.1016/S1473-3099(19)30543-2 PMID: 31657776
6. Goehring A, Lee CH, Wang KH, Michel JC, Claxton DP*, et al.* Screening and large-scale expression of membrane proteins in mammalian cells for structural studies. Nat Protoc. 2014; 9: 2574-85. https://doi.org/10.1038/nprot.2014.173 PMID: 25299155
7. Kim J, Tan YZ, Wicht KJ, Erramilli SK, Dhingra SK*, et al.* Structure and drug resistance of the *Plasmodium falciparum* transporter PfCRT. Nature. 2019; 576: 315-20. https://doi.org/10.1038/s41586-019-1795-x PMID: 31776516
8. Wright DJ, O'Reilly M and Tisi D. Engineering and purification of a thermostable, high-yield, variant of PfCRT, the *Plasmodium falciparum* chloroquine resistance transporter. Protein Expr Purif. 2018; 141: 7-18. https://doi.org/10.1016/j.pep.2017.08.005 PMID: 28823509
9. Sastry GM, Adzhigirey M, Day T, Annabhimoju R and Sherman W. Protein and ligand preparation: parameters, protocols, and influence on virtual screening enrichments. J Comput Aided Mol Des. 2013; 27: 221-34. https://doi.org/10.1007/s10822-013-9644-8 PMID: 23579614
10. Jacobson MP, Pincus DL, Rapp CS, Day TJ, Honig B*, et al.* A hierarchical approach to all-atom protein loop prediction. Proteins. 2004; 55: 351-67. https://doi.org/10.1002/prot.10613 PMID: 15048827
11. Lomize MA, Pogozheva ID, Joo H, Mosberg HI and Lomize AL. OPM database and PPM web server: resources for positioning of proteins in membranes. Nucleic Acids Res. 2012; 40: D370-6. https://doi.org/10.1093/nar/gkr703 PMID: 21890895
12. Dhingra SK, Gabryszewski SJ, Small-Saunders JL, Yeo T, Henrich PP*, et al.* Global spread of mutant PfCRT and its pleiotropic impact on *Plasmodium falciparum* multidrug resistance and fitness. mBio. 2019; 10: e02731-18. https://doi.org/10.1128/mBio.02731-18 PMID: 31040246
13. Acar A, Nichol D, Fernandez-Mateos J, Cresswell GD, Barozzi I*, et al.* Exploiting evolutionary steering to induce collateral drug sensitivity in cancer. Nat Commun. 2020; 11: 1923. https://doi.org/10.1038/s41467-020-15596-z PMID: 32317663
