# Supplementary material for: Evidence for the early emergence of piperaquine-resistant Plasmodium falciparum malaria and modeling strategies to mitigate resistance
Source: PLoS Pathog. 2022 Feb 7;18(2):e1010278. doi: 10.1371/journal.ppat.1010278 (PMC8853508; doi:10.1371/journal.ppat.1010278)
Supplement: Attachment — Submitted filename: PPATHOGENS-D-21-01921_Rebuttal.pdf [file ppat.1010278.s018.pdf]

Point-by-point reply to comments from the Reviewers. Thank you for providing the recent review and thoughtful editorial assessment and for giving us the opportunity to resubmit a significantly revised version that accounts for comments from all reviewers. Please find below our detailed reply, which shows new text shown in blue. Our revised manuscript shows the major changes highlighted in yellow.

Part I: Summary

REVIEWER 1:
The authors have undertaken a retrospective analysis of PfCRT isoforms identified in parasites isolated from patients in 2003-2004 in Yunnan Province, China. In the original study by Cui et al, 24 isolates were typed and assigned as PfCRT sequences of type China A, 7 as China B, 1 as China C, 1 as China D and 1 as China E. In summary, the authors generated transfectant parasite lines with a view to determining whether these PfCRT mutations confer decreased PPQ sensitivity and have a fitness cost. They identified China C as showing reduced sensitivity and used biochemical and molecular modelling studies to try to understand the molecular basis for the reduced sensitivity. Specifically, the authors generated isogenic control and transfectant parasite lines in the Dd2 background. They expressed the PfCRT China E (akin to GB4 + I371R), China B (China E + E75D + A144Y + 138 S220A) and China C (China B + R371I) isoforms.

Reply: We thank the reviewer for this summary of our work.

Comment #1: *Dd2_China C demonstrated a moderate gain of PPQ resistance, compared to the control line Dd2_Dd2. It would be useful to indicate the points of significant difference on* ***Figure 1****. Interestingly, Dd2_China C, which differs from Dd2_China B only in an R371I mutation, did not show a statistically significant difference in PPQ sensitivity compared with Dd2_Dd2.*

Reply: We have now indicated these points of significance in our revised **Figure 1A**. Given that the closest PfCRT isoform to China C is the GB4 isoform, we chose to indicate significance points relative to that line. Significance values to both the Dd2 and GB4 isoforms can be found in **Table S1**. We further discuss the sequence differences between China C and China B below in our Reply to **Reviewer 1 Comment #6**.

Comment #2: *The authors state that "Dd2_China C parasites had distended digestive vacuoles in both the trophozoite and schizont stages, similar to other PPQ-resistant lines, albeit not as pronounced as those in Dd2_Dd2+F145I (****S3 Fig****)." There does not appear to be a panel for Dd2_Dd2+F145I in* ***S3 Fig****. Also, Dd2_Dd2 appears to exhibit a distended digestive vacuole phenotype. Is it mislabeled?*

Reply: We thank the reviewer for this astute observation. In the revised **S3 Fig** we now include a panel for the Dd2_F145I line. Previously it has been shown that the chloroquine-resistant piperaquine-sensitive Dd2_Dd2 line has a moderately distended digestive vacuole in trophozoites but not schizonts. Often the PPQ-resistant lines have a larger digestive vacuole in trophozoites, which persists into schizont. We now clarify these observations in our revised text as follows (starting page 6 lines 169-73): "Of note, the $Dd2^{China\ C}$ parasites displayed distended digestive vacuoles in both the trophozoite and schizont stages, similar to other PPQ-resistant lines, albeit not as pronounced as those observed in either stage of the highly PPQ-resistant $Dd2^{Dd2+F145I}$ line (**S3 Fig**) [8,9,43]. The CQ-resistant PPQ-sensitive $Dd2^{Dd2}$ parasites had mildly distended digestive vacuoles in the trophozoite stage but not the schizont stage, as previously observed [43]."

We also refer to these points in the modified **S3 Fig** legend as follows (page 26, lines 975-6): "$Dd2^{Dd2+F145I}$ has the most distended vacuoles in both stages. Of note, the $Dd2^{Dd2}$ parasites have distended vacuoles in the trophozoite stage only as previously reported [43]."

Comment #3: *The authors measured PPQ uptake by PfCRT-containing proteoliposomes. The China C PfCRT isoform exhibits a similar level of PPQ uptake as the more highly PPQ resistant control, Dd2+F145I. It would be interesting to also examine PPQ uptake by the China B and Dd2_Dd2+A144Y isoforms as this may provide insight into the reasons these very closely related isoforms do not confer resistance.*

Reply: We would like to thank the reviewer for this excellent suggestion. We have attempted on several occasions to purify both of these isoforms but without success. All of our Dd2-like isoforms have been difficult to purify from our heterologous expression system (HEK293 cells), unlike the South American 7G8 isoform for which we were able to obtain sufficient amounts of stable protein to solve its structure by cryo-EM (Kim *et al.* 2019 *Nature*). It should also be noted that we have never been able to see PPQ transport in a sensitive isoform. These points are discussed in our revised text as follows (page 7, lines 223-6): "We were unable to purify the Dd2+A144Y or China B isoforms to perform drug uptake assays with our proteoliposome system. However, we have never been able to detect PPQ uptake with a PPQ-sensitive PfCRT isoform in our assays and thus we would not expect to observe drug transport with either of these isoforms [32]."

Comment #4: The authors modelled the effects of the mutation on the PfCRT structure. They observed differences in the conformation of the cavity, due to the A144Y mutation. The structural modelling analysis fails to give a clear molecular explanation of the sensitivity phenotype. The authors suggest that the A144Y-associated conformational change might underpin PPQ resistance. However, they should comment further on the fact that the A144Y mutation is also present in China B, which is not PPQ resistant.

Reply: In response to this **Comment** as well as **Comments #5 and** #6 below, we have further expanded our Results and Discussion sections to comment on the complex interplay between the A144Y, R371I and T35I mutations. Our revised Results section now reads: "Possibly, the change from the positively charged arginine residue to the uncharged isoleucine in residue in China C allows for improved entry of the positively charged PPQ molecule into the transporter cavity. Modeling cavity electrostatic potentials for the Dd2, Dd2+F145I, China C, and China B isoforms predicted no appreciable differences in cavity, suggesting that differences in PPQ resistance in these isoforms is dependent on cavity conformational changes, rather than cavity charge (**S5B Fig**)."

Our revised Discussion now reads (page 13, lines 428-46): "Molecular dynamics simulations suggest that PPQ resistance is likely more dependent on conformational changes in the PfCRT cavity than on changes in its electrostatic properties (**Fig 3A and 3B; S5 Fig**). China C demonstrated a drastic shift in TM2 and TM7 as compared to Dd2+F145I, which suggests that multiple cavity conformations can enable PPQ efflux. Changes in cavity shape and size observed between the Dd2 and China C isoforms may also explain why the addition of A144Y present on TM3 and facing the central cavity does not alter PPQ susceptibility in Dd2, given that it lacks the E75D, A220S, S326N and T356I mutations found in China C. One *in vitro* study recently examined the roles of the 326 and 356 residues on PPQ resistance [17]. While the 356 residue was found to have no contribution to PPQ resistance, the S326 residue appeared to provide some protection at lower levels of this drug [17]. This study further supports the hypothesis that PPQ transport is dictated by constellations of residue changes as opposed to being driven by a single point

mutation on any given PfCRT isoform. A similar observation was made by Gabryszewski et al. who showed that CQ resistance was only achieved with four or more mutations in PfCRT [21]."

China B and C differ only at position 371 located on the digestive vacuole-facing flexible loop. Even though they share the novel A144Y mutation, only China C is PPQ resistant. Their differences in PPQ susceptibility may result either from an allosteric effect of this variant residue on cavity conformation or from the charge difference between the positively charged arginine and the uncharged isoleucine at residue 371 that might impact drug access to the cavity (**S5B Fig**). The differential impact of these variant residues highlights their complex interplay in conferring drug resistance phenotypes."

Comment #5: *Similarly, the mutation is present in Dd2_Dd2+A144Y (which differs from China C only at T356I) and is not PPQ resistant. What is the predicted effect of the T356I mutation?*

Reply: We would like to clarify that Dd2 + A144Y differs from the China C isoform by four mutations, among which T356I is one (see **Table 1**). The others are E75D, S220A and S326N. The role of T356I in relation to PPQ resistance was recently described in Dhingra *et al.* 2019 *mBio*, as cited above in our reply to **Comment #4**.

Comment #6: *Reside 371 is located at the cavity entrance on the digestive vacuole side. The authors should comment whether changes from Arg to Ile might be expected to facilitate approach by the positively changed PPQ. The authors could use their models to examine the surface charge in this region.*

Reply: We have now addressed the potential role of the 371 residue as noted above in our reply to Comment #4. Molecular dynamic simulations predict no differences in cavity shape or distance between 144Y and either R371 or 371I (**S5 Fig**). This modeling, however, is only suggestive given that the 371 residue is located on an unstructured loop with low resolution in the original cryo-EM structure. We also do not yet have a drug-bound structure to further study potential interactions between this residue and PPQ. We note this caveat in our revised Discussion as follows (page 13, lines 448-50): "Furthermore, there has not yet been a reported drug-bound structure of PfCRT with CQ or PPQ, which would help inform how particular mutations alter drug interactions with the central cavity."

Comment #7: *Do the authors have information about the levels of expression of PfCRT in the different cell lines?*

Reply: We thank the reviewer for raising this point. While we did not do Western blot studies for the lines in this paper, multiple prior Western blot studies from our group have previously reported that edited isoforms do not show appreciable differences in their protein expression levels. We now refer to these studies as follows (page 6, lines 187-90): "While we have not performed Western blot studies with our *pfcrt*-modified lines, we note that multiple prior studies have assessed *pfcrt*-edited lines that differ in their drug susceptibility profiles and have found no detectable differences in PfCRT protein expression levels using Western blot detection [22,44,45]."

Comment #8: *The authors have developed a computational model, which they have used to simulate the effects of fitness and drug sensitivity differences on parasite burden during and after treatment with combinations of PPQ and CQ. This model generates interesting predictions but may be oversold in terms of its usefulness. For example, the authors' conclusion that a combination of PPQ and CQ is effective at clearing PPQ-resistant infections is a very optimistic*

view of the simulations, which showed that resistance is predicted to arise in all scenarios other than the logistically difficult and potentially toxic scenario where patients receive 3 days of simultaneous high-dose PPQ and CQ treatment.

Reply: We have now toned down the conclusions of this section and included more discussion on the high dosing levels, as stated below.

First, and in response to **Reviewer 2 Comment #3** we clarify that the modeled high doses of PPQ and CQ are close to or below their published C_{max} values, now noted in the results section (starting page 9, lines 312-5): "Low- and high-dose drug concentrations remain at or below the concentrations observed in patient samples *in vivo* [48,51,52]. However, it should be noted that the high dose concentrations are close to peak serum concentrations for PPQ (539 ng/mL, 539 nM) [53] but well below those observed for CQ (376 ng/mL, 1.18 uM) [48]."

Second, we have now run the simulations where PPQ and CQ are dosed sequentially rather than simultaneously. The results for both outcomes were nearly identical. In our revised manuscript we now add the results of simulating sequential drug administration to our revised **Table 2** and our **Tables S6, S7, and S8**. We have also updated the text to refer to these sequential drug dosing simulations.

Third, we have modified the last paragraph of our Results section to clarify that these experiments are theoretical and provide an intriguing new hypothesis to further purse (page 12, lines 396-400): "In the high-dose regimens, infections with $Dd2^{Dd2+F145I}$ or $Dd2^{Dd2+I218F}$ treated with sequential or simultaneous PPQ+CQ dosing eliminated all parasites with no predicted emergence of new variants (**Table 2; S6 Table**). Taken together, these modeling data provide theoretical evidence that the addition of CQ to a high-dose PPQ regimen might be an effective strategy that is worth further exploration, as the combination was predicted to effectively treat PPQ-resistant infections and suppress the *de novo* within-host evolution of modeled variants."

Our revised Discussion also refers to this as follows (page 14, lines 481-2; see also our reply below to **Comment #9**): "We note that the high-dose levels in the model are on the higher end and within the normal range of PPQ and CQ blood concentrations observed with standard dosing, respectively [51,65]."

Comment #9: *In the discussion, the authors note that there is little evidence of cardiotoxicity associated with PPQ treatment. However, it should be noted that it can prolong the QT interval and may increase the toxicity of chloroquine. The conclusions from this section need to be more moderate.*

Reply: We thank the reviewer for these important comments. Our revised Discussion now states that the QT prolongation has been studied for the drugs individually and would need to be studied more extensively if the combination were to be used. The new text now reads (age 14, lines 482-7): "Individually, CQ and PPQ have been used extensively. Both drugs have been demonstrated to prolong the QT interval, yet neither have led to clinically significant cardiac events [66,67]. It remains to be determined, however, whether the combination of the two medications either dosed simultaneously or sequentially would alter cardiotoxicity. Future studies would also be required to determine tolerability, pharmacokinetics/pharmacodynamics, and safety profiles of the combinations."

Comment #10: Similarly, the title suggests that the manuscript describes a path for evolution of PPQ-resistant parasite. The currently presented simulated data are not sufficiently strong to support this conclusion.

Reply: We thank the reviewer for this comment and have now revised our title as follows (page 1, lines 1-3): "Evidence for the early emergence of piperaquine-resistant *Plasmodium falciparum* malaria and modeling strategies to mitigate resistance".

REVIEWER 2:

The submission by Small-Saunders and colleagues is a detailed exploration of the role of the PfCRT protein in the function of chloroquine (CQ) and piperaquine (PPQ), both related 4-aminoquinoline antimalarials. The evolution of resistance to these drugs is a major issue in the control of malaria caused by Plasmodium falciparum. It has been observed in the field that resistance to these two drugs seems to have a reciprocal relationship, and distinct mutations in the pfcrt gene may be responsible. A previous paper from many of the same authors (reference 30) described the structure of key parts of the PfCRT protein from some CQ and PPQ resistant parasites, and compared the binding and transport properties of these molecules. The current submission builds on these technical advances, but changes the focus to explore in detail the properties of a set of parasites that carry versions of PfCRT that differ from one another in single amino acids. The goal is to use a coordinated set of these lab-based approaches to illuminate the possible evolutionary history of PPQ resistance. They then use these well-defined parameters that they have measured to consider various ways that the CQ treatment might be used in the field to "steer" evolution from selecting PPQ resistance to an evolutionary dead end instead. This paper is important. CQ resistance is almost universal in many Pf endemic regions and intensive use of PPQ has been observed repeatedly to favor evolution of CQ susceptibility in those populations. PPQ resistance is especially pervasive in the north-eastern parts of the Greater Mekong Region, but a recent publication has also uncovered declines in PPQ responses in Uganda, as well, so the manuscript addresses an urgent and growing problem in malaria treatment.

Specific

- The manuscript compares a set of 12 highly related parasites with edited genotypes that differ by 1 or more mutations in the pfcrt gene, and/or differences in the canonical PfCRT (SVMNT compared with CVIET residues 72-76).

- The rate of growth in culture without drug(s) and their susceptibility to a range of drugs was measured carefully, and compared to establish these parameters for each cultured line.

- Then, capitalizing on prior work of the group, the biochemical and structural properties of some of the lines are compared directly to try to define differences in binding of CQ and PPQ to the PfCRT proteins and the transport of each drug in a standardized system to estimate transport capacities.

- The solved structures of the PfCRT proteins expressed by the various lines are then modeled and compared to define derangement(s) in the binding pocket of each protein that could allow these differences to explain the CQ and PPQ responses in the cultured lines.

- These data sets are then reported in the figures and tables (and especially within the Supplementary files).

This part of the paper is quite overwhelming, but the authors have provided a number of tables with the genotypes of each cultured parasite line (which I kept visible on my second monitor) and the figures are clear and well defined. The systematic comparisons make it possible to follow the logic that considers the relative importance of particular mutations to differences in growth in the lab with and without drug treatment and so on.

The collection of the data in the manuscript is then entered in a newly created model to compare outcomes of treatments with PPQ and CQ of each of the 12 lines used. The idea is to test the potential success in patients infected with a specific genotype of parasite comparing various combinations and timing of PPQ plus CQ "treatments". A de novo model was created and populated with the data trove reported in the first part of the manuscript. The code and the data are both publicly available in the Supplement, and the authors encourage others to use their model and data to try various other parameters.

The goal seems to be to incentivize clinical teams to consider testing combinations of CQ and PPQ as "triple ACTs" with the goal of possibly adding CQ + DHA PPQ to the triple ACTs currently under clinical assessment in both SE Asia and in Africa. That is suggested at the end of the discussion along with other kinds of comparisons that others might want to explore.

Reply: We would like to thank the reviewer for this detailed and positive summary of our study and for highlighting this as important.

Comment #1: *The authors point out that fitness of particular parasite genotypes is not (yet ?) considered in the model, since there is no lab-based parameter except growth in culture. One genotype of PfCRT (CVIET common in parts of Asia and Africa does seem to be rapidly cleared when not under CQ pressure). But the complexity of parasite growth in humans doesn't allow any standardized metric, though the authors do mention that immunity to parasites can't really be studied on a population basis.*

Reply: We agree with the Reviewer that parasite growth and infection within the host, as well as how transmission plays into shaping parasite populations, is very difficult to model. In response to this Comment as well as **Comment #2** and **Reviewer 3 Comment #15** we have added a new paragraph that discusses challenges and caveats of our study (page 15, lines 496-512): "While the simulation data provide an intriguing framework to assess novel combination treatment regimens, certain limitations of the study should be acknowledged. First, all IC_{50} and modeling data presented herein were based on *in vitro* data of *pfcrt*-edited parasites in the Dd2 background. While this allows an assessment of the *pfcrt* contribution, it does not take into account how past or current circulating parasite backgrounds can affect drug susceptibility or parasite fitness. Further studies will be needed to assess how combination therapies are influenced by the parasite background, including *plasmepsin* copy number and changes in *pfmdr1* and *pfk13*. It should also be noted that *in vitro* data and simulation models cannot encompass all the complexities that occur within the individual host, including patient co-morbidities, variations in drug metabolism, and intrinsic susceptibility to malaria infection. Notably, our model does not incorporate immune responses that are of particular relevance to high-transmission regions in Africa. However, given the low transmission rates and sparse immunity in SE Asia [49], we would not expect this to substantially alter our simulation outputs for this region. Future studies could use data from these simulations as a starting point for more in-depth *in vivo* studies including in murine infection studies. Our simulations also did not include transmission dynamics, which would be expected to impact the evolution of parasites at the population level. Despite these caveats, our simulations provide a computational tool to explore how parasite density, amino acid mutations, drug pressure, dosing regimens and fitness can combine to shape the evolutionary landscape of parasite populations in the host."

Comment #2: *They do begin the paper's background with a Chinese Pf parasite that carries low level resistance to PPQ that apparently evolved under use of PPQ as a monotherapy early in China. Still, I would wish for at least a short paragraph at the end of the discussion that*

acknowledged the complexity of outcomes in real patients under treatment, especially outside of the very low transmission that is now the case in SE Asia. That is not a criticism of the current paper- it is a tour de force, to say the least. But, setting expectations for the CQ+PPQ approach in areas of high transmission where many patients carry parasites with different genotypes is more than challenging.

Reply: We thank the Reviewer for this comment and have incorporated this suggestion in our response to **Comment #1** above.

Comment #3: *Also, they mention that the safety of combining 2 4-aminoquinolines with similar potential toxicity is a worry and has been studied and has not been a real threat to patient safety. However, the levels used in the high concentration version of the model are very high indeed. I was unable to find quickly the maximum allowed exposure to PPQ or CQ. The tolerability of the high concentrations that were most effective in the model might be worth clarifying.*

Reply: These are important observations. We refer the Reviewer to our detailed response to similar Comments raised by **Reviewer 1 (Comments #8 and #9).**

Comment #4: *A very small detail- in most of Africa, the predominant parasites are currently CQ sensitive. That is not so much a result of the withdrawal of CQ, but rather that the currently used ACT is artemether- lumefantrine, and that drug has over time selected the CQ sensitive allele repeatedly. Lumefantrine is a somewhat related molecule to CQ and PPQ, so that is worth pointing out as a possible current example of these kinds of evolutionary selection in the real world.*

Reply: Our revised Discussion now includes this reference to lumefantrine as follows (page 14, lines 469-71): "Of note, the partner drug lumefantrine selects against CQ-resistant PfCRT isoforms, and is associated with the return of CQ sensitivity and wild-type *pfcrt* in Africa [15,62]."

Comment #5: *In sum, the paper is excellent and uses a wide range of technical approaches to test a well-defined set of Pf parasites and apply what they learned to the possible application of PPQ and CQ treatments in people. It may stimulate studies in people to test whether similar responses might be possible. That might work in SE Asia where the complexity of human infections is low. The paper makes a reasonable case that it could be worth a try.*

Reply: We thank the Reviewer for this enthusiastic summary.

REVIEWER 3:
Small-Saunders et al provide exciting new data on the role of pfcrt variants in mediating resistance of P. falciparum to the antimalarial drug piperaquine. Several improvements in the manuscript are needed for the data to be presented appropriately and clearly.

Reply: We thank the Reviewer for this positive endorsement of our work and for the helpful comments listed below.

ABSTRACT
Comment #1:
Line 32: multi-drug resistant parasites have not "swept across Southeast Asia". In fact,
- the *P. falciparum* populations in the Mekong countries are crashing; you are describing (in this assertion) a remnant population sliding towards elimination.
- these parasite genotypes from Cambodia have not established themselves in Myanmar

- in other SE Asian nations with a reasonable *P. falciparum* burden piperaquine resistance is not reported (e.g. Indonesia).
- your own data show the Yunnan genotype is of independent origin and has NOT swept in from Cambodia, nor has it swept southwards.
- China has now eliminated Pf and Pv malaria, so these Yunnan genotype parasites are presumably extinct (or a residual population may be extant on the Myanmmar side of the border). Please tone down this unwelcome polemic.

Reply: Our revised Abstract has removed the word swept" and now reads (page 2, lines 32-33): "Multidrug-resistant *Plasmodium falciparum* parasites have emerged in Cambodia and neighboring countries in Southeast Asia"

INTRODUCTION

Is it REALLY necessary to introduce yet another three-letter abbreviation (ABS)? This is simply confusing, as it is non-standard to my knowledge. IE (infected erythrocyte) or iRBC are more common, but then in a world of digital publishing why not just write out in full or simply use "parasites" (having defined at outset that this refers to blood-stage asexuals)? Especially since it only occurs a handful of time apart from the Methods section.

Reply: We have now replaced all the "ABS" abbreviations with asexual blood stage.

Comment #2: *Line 70-71: CQ was not directly replaced by ACT in most countries, but by SP or SP-AQ or SP-CQ or mefloquine.*

Reply: Our revised text (page 3, line 71) has replaced "directly" with "ultimately".

Comment #3: *Line 74: see comment #1.*

Reply: Our revised Introduction now states (page 3, lines 73-4): "However, delayed parasite clearance after ART treatment is now present throughout SE Asia."

Comment #4: *Lines 85-94: I can understand that it is helpful intra-group shorthand to label haplotypes by a corresponding laboratory cell-line ID (GB4, Dd2 etc) but really, for the majority of the field unfamiliar with these lines (or their geographical and temporal origins) it is more precise and informative to use the full pfcrt locus haplotype, As our new understanding of the gene indicates an ever more complex pattern of variation, this becomes even more important. How do you describe a parasite, genotyped directly from a patient, that is "... a bit GB4 with some 7G8 thrown in at the carboxy terminal end"?*

The approach I am advocating is less succinct, as precision often is, and requires careful definition to start with, but is preferable in my view. This would comprise up to at least 13 amino acid positions if deployed in full (codons 10, 24, 72, 74-76, 93, 145, 218, 220, 271, 326, 371). The wild type would be: QDCMNKFTIAQNR. The imaginary "fully variant" haplotype would be: KYS(I/M)ETISFSESI. The authors may know of other variant positions that could be added to this. Some authors, in providing haplotypes in this form, use bold font and/or underlining to denote variant positions. I hope the authors will consider this approach, much more genetically rigorous, as the new norm in the Fidock lab, which the whole field looks to for establishment of the benchmark in crt studies. Of course, it may be decided to just stick with your Table 1 showing the haplotype for each parasite line or patient-derived genotype mentioned in the paper, and then continuing with the shorthand, but this just kicks the can a very short distance down the lab in my

view. The need you have found to add positions to your superscript modifiers in Table 1 perfectly illustrates the problem.

Reply: We would like to thank the Reviewer for this thoughtful suggestion for naming *pfcrt* alleles. While we agree that it would be more genetically rigorous, we would prefer to continue with our current nomenclature in order to make this publication similar to our prior studies. We also worry that describing the full haplotype as a superscript would make it very hard for readers to follow and also allow for potential errors. Another concern is that future work will undoubtedly reveal yet more PfCRT mutations, which would not be captured by already defining a select number of residues that comprise a haplotype. We will continue to search for ways to be readily comprehensible and fully accurate with the ever-expanding list of haplotypes, but for now feel that Table 1 remains the most suitable for this manuscript.

Comment #5*: Line 97: In this example you could write: "These mutations all evolved from the CQ-resistant PfCRT haplotype CVIET at codons 72-76 (refs)."*

Reply: see above reply to **Comment #4**. The CVIET cluster of mutations is observed in several haplotypes, including GB4, Dd2, FCB and Cam783. We therefore feel it is preferable to specify Dd2 and GB4, as these are the most relevant PfCRT haplotypes to refer to in our study.

Comment #6: Line 109: briefly define "polytopic" for readers not familiar with trans-membrane protein nomenclature.

Reply: We have now removed the term "polytopic" and revised this sentence as follows (page 4, lines 109-11): "PfCRT is a digestive vacuole protein with multiple transmembrane domains and a central negatively-charged cavity comprised of four pairs of antiparallel helices, as determined using cryo-electron microscopy [32]."

RESULTS

Comment #7: Line 136: Readers unfamiliar with Dd2 would benefit from a brief description of its pfmdr1 status here (mutations, amplification status).

Reply: We have updated the results section to now include the *pfmdr1* information on Dd2 as follows (page 5, lines 136-8): "The Dd2 line used herein expresses 2 copies of *pfdmr1* that encodes the N86Y mutation, as well as a single copy of the *plasmepsin II* gene [8,42]."

Comment #8: *Lines 136-139: this passage would be much easier to read if you just used the full haplotypes, instead of phrase like "China E + E75D + A144Y + 138 S220A"*

Reply: We recognize that this is not straightforward, but the sentence does allow a logical flow: "PfCRT China E (akin to GB4 + I371R), China B (China E + E75D + A144Y +S220A), or China C (China B + R371I) isoforms." We worry that listing long haplotypes would allow have its own difficulties in not readily highlighting the residues that were variant and prefer to keep these haplotypes listed as per the original report from the group of Liwang Cui and in keeping with prior literature describing GB4 (Yang Z *et al.* 2007 *Trop Med Int Health*; Sa *et al.* 2009 *PNAS*; Dhingra *et al.* 2019 *mBio*).

Comment 9: Nice data in Fig. 1. Would be best to have the legend sitting outside the graph boundary (to the right?). It is helpful that the line names are ordered from most-to-least susceptible, but this only holds for the final concentration.

Reply: We thank the reviewer for this positive assessment of the data in **Figure 1.** We have now modified this figure in response to this comment and that of **Reviewer 1**. The legend is now placed outside the graph boundary and we have also added points of significance between Dd2_China C and Dd2_GB4 parasite lines.

Comment #10: Lines 154-162: results for standard 72h EC50 estimates are interesting and fit well with recent findings of the Cui lab (ref 36). This directly links the F145I allele to the bi-phasic dose-response curves seen in Cambodian studies. Does this render the PSA survival assay unnecessary in parasite populations lacking this parasite phenotype? Are EC50 plus EC90 estimates enough to characterize most parasite isolates? The China E result is fascinating - implying that loss of the aminoquinoline resistant haplotype STI at pfcrt codons 326, 356 and 371 generates a benefit in 72h PPQ dose-response assays compared to the Dd2 haplotype (and in EC50 reduction Fig. 1A). Such parasites are present in Africa (e.g. Sudan and Tanzania in https://pubmed.ncbi.nlm.nih.gov/25253286/) and need investigation perhaps?

Reply: Here, the Reviewer refers to our finding that the 72 hr dose-response assays identified elevated IC_{50} and IC_{90} values only with the isogenic Dd2 parasites expressing PfCRT Dd2+F145I or China C. These were also the only lines demonstrating an elevated survival rate in the PSA, with the Dd2+F145I line showing the highest level of resistance in both assays. Our prior studies with the PPQ-resistant isoforms Dd2+T93S, Dd2+I218F, Dd2+M343L or Dd2+G353V also revealed resistance in the PSA assays and based on their increased IC_{90} values (Ross *et al.* 2018 *Nature Comm*; Dhingra *et al.* 2019 *Lancet Infect Dis*). All of these mutant lines showed biphasic dose-response curves, with the more extreme biphasic responses observed in lines with the highest PSA survival levels. In the study from the Cui lab (original ref. 36), there was no association between IC_{50}, IC_{90}, AUC and PSA values. However, very few parasites (7 of 120) showed a PSA value >10% and importantly, only a single isolate harbored a PPQ resistance-associated PfCRT mutation (H97L) and none had multicopy plasmepsin II/III. Those data provide evidence that these parasites were almost all PPQ-sensitive, with very low to very low IC_{50} and IC_{90} values.

The Reviewer here poses the interesting question of whether IC_{50} and IC_{90} measurements suffice in areas that lack the biphasic response seen with parasites expressing the high-grade resistant mutation Dd2+F145I, or that would be seen also with other resistance-conferring mutations such as T93S, I218F or G353V. Based on our understanding to date, we would argue that 72 hr dose-response assays are sufficient to profile parasites in areas where these novel PfCRT mutations or multicopy plasmepsins have not emerged. The PSA is nonetheless very useful to quantify the degree of resistance, as very high-grade resistance such as that observed with the F145I variant cannot be accurately measured at the IC_{50} or IC_{90} level given the minimal impact on IC_{50} values and the biphasic dose-response curve observed at higher PPQ concentrations.

Our revised Results now refers to this as follows (page 5, lines 64-7): "Based on the dose-response profile and PSA data, the China C allele did not confer the high-level PPQ resistance we have previously observed with contemporary PfCRT variants. These isoforms, including Dd2+F145I, Dd2+T93S, Dd2+I218F or Dd2+G353V, have been more recently detected in Cambodia and, unlike China C, display biphasic PPQ dose-response curves [8,9]."

In our revised Discussion we now add (page 12, lines 404-6): "Herein, we characterize a novel PfCRT isoform (China C) from Yunnan Province, China, that confers moderate resistance to PPQ, based on elevated PSA survival rates and IC_{90} values"

We thank the Reviewer for pointing out the China E data. Our dose-response data showed that, in comparison to the related GB4 allele the China E isoform showed a slight increase in the PPQ IC90 (S2 Fig). There was however no shift in the PPQ IC50 or the PSA survival data. The China E line also showed a partial loss of CQ resistance compared with GB4 (**Fig 1B, C**). This China E allele is also closely related to a small number of African isolates that express the same CVIET (72-76) and NIR (residues 326, 356 and 371) haplotype, as reported by Gadalla *et al.* 2015 *J Antimicrob Chemother* (PMID citation provided by **Reviewer 3**). We note however that China E was not identical as it harbored the Q271E mutation whereas the African isolates had wild-type Q271. Nonetheless, given that GB4 is quite prevalent in Africa and is only one mutation away from China E, we agree that further investigation of GB4-like variants in Africa is merited. Indeed, this is an active area of investigation in our lab.

Our revised Introduction now includes the Gadalla reference as follows (page 3, lines 88-9): "GB4 has now become the predominant mutant PfCRT haplotype in Africa [16, 17]. Our revised Results also now incudes (page 5, lines 161-2): "Dd2$^{China\ E}$, which lacks the GB4 R371I mutation and differs from China C at four residues, was also PPQ-sensitive."

Comment #11: Lines 164-174: Very interesting and important data. As a range of drugs are used in the study, and more than one assay format, the authors should consider switching from the "IC50" nomenclature to the EC50 form (effective concentration); this is becoming more widely accepted for *in vitro* susceptibility studies in the antimalarial field. The reasoning is that for most assays it is not possible to ascribe the measured effect to inhibition of growth alone, which would be to ignore variable cytotoxic effects which are drug-dependent in their relative importance.

Reply: We agree with the reviewer that the EC_{50} form is starting to become more widely accepted in the field. Nonetheless, in order to keep this study comparable to other studies from our lab and most others we would prefer to continue using the IC_{50} nomenclature at this time. We are not fixed on this however and can make this change if deemed imperative.

Comment #12*: Lines 211-232: This interesting analysis relies on the cryo-EM structural solution for the SVMNT allele (7G8) at codons 72-76. Are the differences from CVIET (Dd2) relevant to understanding the outputs? Are there some caveats due to this?*

Reply: The Reviewer makes a good point, and our revised Discussion acknowledges the differences in the solved structure and the molecular dynamic simulations as follows (page 13, lines 446-50): **"**One caveat to the presented molecular dynamics simulations, however, is that they were performed using the Dd2 isoform for which the structure has not been solved. Dd2 differs from the solved 7G8 structure at positions 72, 326 and 356. Furthermore, there has not yet been a reported drug-bound structure of PfCRT with CQ or PPQ, which would help inform how particular mutations alter drug interactions with the central cavity."

Comment #13: *Pages 7-10: These fitness/evolutionary modelling analyses are of interest, particularly the putative interplay between PPQ resistance and CQ susceptibility. However the term "evolutionary" should probably not be used, because the single most important event in the true fitness of a parasite population is transmission to the mosquito. You are really considering blood-stage fitness in isolation of transmission potential. As P. falciparum gametocytes are dependent on Hb catabolism for nutrition until at least stage IIb, and have an active digestive vacuole susceptible to CQ and AQ in vitro and in vivo, this need to be acknowledged and woven into the overall argument. You do not present any data on how transmissible the variants studied are. (This needs acknowledgement, but I am not suggesting this is a flaw in what is presented thus far.)*

Reply: We have now clarified "evolutionary" in the text to say "within-host evolution", as we are assessing how the parasite population would evolve in the host in response to drug pressure. We also acknowledge that evolution on a population-based level would need to include a discussion of transmission, which we note in the revised manuscript in the Results section as follows: (page 9, lines 301-3): "Given that we were assessing within-host evolution of parasite *pfcrt* alleles, we did not include parasite transmission dynamics in this model. Such data would be necessary to make conclusions on a population evolution basis [50]."

Our revised Discussion now includes (page 15, lines 508-9): "Our simulations also did not include transmission dynamics, which would be expected to impact the evolution of parasites at the population level. "

DISCUSSION

Comment #14: *Lines 417-418: although triple ACT as explored by Dondorp and colleagues hold some promise, they do not overcome the major issue with our current ACT regimens - three days of artemisinin treatment is insufficient to clear parasites. Would not a better application of your data be the sequential administration of two ACT (DHA-PPQ followed by AS-AQ seems the best choice to me) which provides the "evolutionary trap" you require AND gives 6 days of artemisinin. This approach (see https://pubmed.ncbi.nlm.nih.gov/29082016/) has the advantage of using regimens already licensed and available for trials immediately. Given the low numbers of cases in areas blighted by DHA-PPQ resistance, adherence this special 6-day regimen could be feasibly managed in my view. The availability of the AS-AQ regimen already trumps your arguments on the advantages of CQ, as adding the three days of additional artemisinin is in my view crucial - note that it is well known that K13 variant parasites are fully susceptible in vivo to 7 days artemisinin monotherapy.*

Reply: The Reviewer raises an interesting point. Our monodesethyl-amodiaquine data, however, showed no significant gain of amodiaquine potency against our PPQ-resistant isogenic lines (S4C Fig). In earlier studies, we had observed a slight increase in amodiaquine potency with some PPQ-resistant PfCRT variants, but not all [Dhingra, 2019 #4;Ross, 2018 #1]. Further evaluation of PfCRT variants is required before we are confident in stating that PPQ and amodiaquine could constitute an evolutionary trap. Our revised Discussion now cites the paper mentioned above that describes a trial protocol to explore sequential treatments with double ACTs including an artesunate-amodiaquine arm (https://pubmed.ncbi.nlm.nih.gov/29082016), as follows (page 16, lines 525-7): "For instance, this could be expanded to assess treatment regimens with amodiaquine or mefloquine, or to model PfK13 mutants after ART or ozonide treatment [77,78]."

Comment #15: *The authors need a short summary in the Discussion of study weaknesses: use of the Dd2 background (an ancient workhorse), modelling based on a structure from a different allele of pfcrt, evolutionary fitness explored in the absence of a transmission component etc.*

Reply: We have now included this summary, as detailed in our replies above to **Reviewer 2 Comment #1** and **Reviewer 3 Comment #12**.

Part II – Major Issues: Key Experiments Required for Acceptance
Please use this section to detail the key new experiments or modifications of existing experiments that should be absolutely required to validate study conclusions.

Generally, there should be no more than 3 such required experiments or major modifications for a "Major Revision" recommendation. If more than 3 experiments are necessary to validate the study conclusions, then you are encouraged to recommend "Reject".

Reviewer #1: *The insights into the molecular basis for why China C exhibits decreased PPQ sensitivity while closely related isoforms are limited. Analysis of PPQ transport in PfCRT isoforms for China B and Dd2_Dd2+A144Y would strengthen the manuscript.*

Reply: We fully agree with the Reviewer about the benefit of obtaining PPQ transport data with these isoforms. However, as stated above in our reply to **Reviewer 1 Comment #3**, we have been repeatedly unable to obtain sufficient amounts of intact, pure protein from these isoforms expressed in HEK293 cells to perform these transport studies. Our reply to that Comment lists the changes that we have made to the manuscript to address this point.

Reviewer #2: *At least some thoughts on how the lab data relate to parasite growth, actual exposure to the two drugs, especially PPQ, and drug responses would make it easier for readers from outside malaria to assess the applicability of the lab data to the field would be very useful, but is not required.*

Reply: Our revised manuscript incorporates additional discussion of these points, as listed above in our response to **Reviewer 1 Comment #9, Reviewer 2 Comment #1,** and **Reviewer 3 Comment #13.** We also expand on clinical correlations to our in vitro data as follows (starting page 9, lines 308-13): "'Low-dose' treatments corresponded to initial blood concentrations of 200 nM PPQ and/or 125 nM CQ. These concentrations were chosen based upon *in vitro* resistance thresholds that had been demonstrated to correlate with clinical resistance [7,28]. 'High-dose' treatment regimens corresponded to initial concentrations of 400 nM PPQ and/or 250 nM CQ. Each treatment was simulated as a once-daily dose for three consecutive days. Low- and high-dose drug concentrations remain at or below the concentrations observed in patient samples *in vivo* [48,51,52]."

Reviewer #3: None.

Part III – Minor Issues: Editorial and Data Presentation Modifications

Please use this section for editorial suggestions as well as relatively minor modifications of existing data that would enhance clarity.

REVIEWER #1:

Comment #1: *The authors refer to a report from 2019 in support of their statement that "More recently, resistance has also emerged to the ACT partner drug piperaquine (PPQ), leading to >50% treatment failures with this combination across the Greater Mekong Subregion". That study found ~50% treatment failure rates in a couple of areas. The authors should provide additional references to more recent studies or modify their statement.*

Reply: We have now revised this sentence to moderate the language and also introduce an comprehensive recent review as follows (page 3, lines 73-6): "However, delayed parasite clearance after ART treatment is now present throughout SE Asia." More recently, resistance has also emerged to the ACT partner drug piperaquine (PPQ), leading to up to 50% treatment failures with this combination in some areas of the Greater Mekong Subregion [4,5]."

Comment #2: *PSA should be defined at first use.*

Reply: We thank the reviewer for pointing out that we had not defined PSA prior to using this abbreviation. We have now updated the text accordingly (page 5, lines 145-6).

Comment #3: *It would be useful to indicate the points of significant difference between Dd2_China C and Dd2_Dd2 on Figure 1.*

Reply: We have now modified **Figure 1** in response to this comment and **Reviewer 3.**

Comment #4: *The China C isolate (China B + R371I) was identified in only 1 of 34 isolates that were typed in the study led by Cui. The authors should discuss why PPQ R parasites were so rare in Yunnan despite DHA-PPQ being widely used.*

Reply: Thank you for this astute observation. We have now added a section in the Discussion to address this as follows (page 14, lines 457-62): "Interestingly, the PPQ-resistant China C isoform was only 1 of the 34 isolates identified in the original study [40]. Multiple factors likely contributed to the lack of PPQ-resistant parasites in the study population despite the widespread use of DHA+PPQ. First, during the sample collection period PPQ had been discontinued as a monotherapy ~20 years prior leading to selection of PPQ-sensitive *pfcrt* alleles [55]. Second, despite DHA+PPQ being utilized at that time, ART resistance was still relatively contained, resulting in less selection pressure on PPQ."

REVIEWER #2:

Comment: *The figures are very busy, to say the least. The figure legends could be improved and that might help, but the many different kinds of lab-based assessments that they used will be a challenge for many readers. I'm not sure that separating the figures into multiple more figures would help.*

Reply: We agree that the figures have significant amount of data in them, which can lead to confusion. We found that the previous **Figure 2**, which included transport data and molecular dynamic simulations was the densest and have now separated it into new **Figure 2** and new **Figure 3**. The remaining figures we felt could be left as they were.

REVIEWER #3:

See section I.

Reply: Our replies are listed above.
